# On the Structural Properties of Discrete-Time and Sampled-Data Hamiltonian Dynamics

**DOI:** 10.3390/e28090967

**Published:** 2026-08-29

**Authors:** Salvatore Monaco, Dorothée Normand-Cyrot

**Affiliations:** 1 Dipartimento di Ingegneria Informatica, Automatica e Gestionale Antonio Ruberti, Sapienza University of Rome, 00185 Rome, Italy; salvatore.monaco@uniroma1.it; 2 Laboratoire des Signaux et Systèmes, CentraleSupelec, Université Paris-Saclay, 91190 Gif-sur-Yvette, France

**Keywords:** port-Hamiltonian modeling, discretization and numerical methods using port-Hamiltonian framework, mathematical methods using port-Hamiltonian formulations

## Abstract

While continuous-time Hamiltonian dynamics are naturally energy preserving with a symplectic flow, their discrete-time counterparts enhance either geometric or energy preservation properties, but rarely both within a unified framework. It is the object of this paper to more deeply investigate this question. In both linear and nonlinear settings, necessary and sufficient conditions characterizing discrete Hamiltonian dynamics that are conservative and symplectic are derived. The relationship with exact sampled models of continuous-time Hamiltonian dynamics are investigated, showing that such models, that preserve both energy and symplectic structures, do not generally fit into the proposed canonical form. Generalized Hamiltonian structures are, thus, introduced. On these bases, Hamiltonian integrators that preserve both the energy and the symplectic structure up to a prescribed order in the sampling period, are constructed. Some simulations on nonlinear test cases illustrate the theoretical findings.

## 1. Introduction

The idea of representing dynamical configurations through conserved quantities, a feature shared by a wide range of observable phenomena including oscillatory behaviors, lies at the core of the Hamilton’s intuition. Within this framework, the evolution of the system is characterized by the preservation along system trajectories of an energy like scalar function, H(x), referred to as the Hamiltonian. The capability of influencing the system evolution while either enforcing or relaxing the constraint imposed by H(x) has led, in the control literature, to the generalization of the Hamiltonian formalism to the so-called port-controlled Hamiltonian (PcH) structures [1]. These structures are fundamental to design methodologies based on equilibrium shifting and energy shaping. This framework nowadays constitutes a well-established chapter of control theory (see [2,3] and the references therein).

The present work is located within the broader framework of modeling Hamiltonian dynamics in discrete time that specifically satisfy the two fundamental conservative and symplectic structures. The underlying motivation is that, in continuous time, these two properties are closely related: under the usual assumptions, requiring one of them leads to the other one. In discrete time, by contrast, there is no general definition of Hamiltonian dynamics that simultaneously enhance both properties, except in the special case of quadratic Hamiltonians.

For this reason the extension of Hamiltonian formulations to the discrete-time setting has been hindered by a fundamental uncertainty regarding the appropriate definition to be adopted, leading to alternative formulations that can be tentatively classified. A first significant body of work set the study on discrete spaces focusing on the preservation of the geometric and, in particular, symplectic structure [4,5,6,7,8]. Inspired by the continuous-time setting, a significant part of the literature is dedicated to integration schemes preserving the structures [9,10,11,12]. In the spirit of a variational approach, important advances are performed exploiting the discrete-gradient-based representation proposed as a mean to recover energy preservation [13,14,15,16,17]. More recently, this body of representations motivates the interest of the control community to develop energy-based control methodologies for discrete-time or sampled-data systems in terms of passivity or energy shaping [18,19,20,21,22,23,24,25,26].

Within this framework, we discuss Hamiltonian representations for discrete-time dynamics and dynamics under sampling, in the nonlinear and linear cases, for dynamics with no control input.

In Section 2 and Section 3, we investigate the symplectic property for a recently introduced class of discrete-time Hamiltonian dynamics based on the discrete gradient, which is conservative by construction. Theorem 1 identifies the conditions under which a symplectic structure is also ensured. These conditions are rather strong and appear to be closely related to the quadraticity of the Hamiltonian. In fact, in the linear setting, both properties are naturally satisfied and the interplay between implicit and explicit representations characterized by infinitesimally symplectic and symplectic matrices, respectively, is clarified. This is similar to the correspondence between the differential form of Hamiltonian dynamics and its flow. On these bases, a necessary and sufficient condition specifying a discrete-time Hamiltonian matrix is described in Theorem 2.

A second issue that cannot be neglected concerns the relationship between such discrete-time models and those issued from sampling their continuous-time counterparts. It is immediately clear that the exact sampled dynamics inherits the relevant structural properties of the underlying continuous-time Hamiltonian one: the sampled evolution is conservative and symplectic (Proposition 7). Theorems 3–5, which reformulate the results in [16], show that the exact sampled dynamics admits a discrete-time Hamiltonian representation that we refer to as “generalized form”. The term reflects the fact that the canonical matrix *J* is replaced by a skew-symmetric matrix, denoted by J(δ) in the linear case and J(δ,x) in the nonlinear case. Finally, exploiting the representation of the solutions through series expansions, one characterizes computable approximate forms of sampled Hamiltonian dynamics. A third order symplectic and conservative Hamiltonian form is described in Theorem 6, while a hierarchy of higher-order approximate symplectic and conservative Hamiltonian forms are described in Theorem 8 in the linear case. In this way, the perspectives of these integrator methodologies move towards numerical analysis or data scientists’ interests. However, even if these devices may represent extensions to the celebrate midpoint or symplectic integrator scheme, a deep comparative investigation is necessary regarding qualitative or computational performances. This goes beyond the scope of this paper.

Simple examples are reported to compare the proposed models with more classical midpoint or first-order approximation schemes.

The paper is organized as follows. The Introduction ends with some notations. Section 2 develops the algebraic framework underlying the variational aspects behind the discrete-gradient formalism. Section 3 introduces a class of discrete-time Hamiltonian forms and analyzes its properties of conservativeness and symplecticity. The linear case is specified. Section 4 addresses the case of sampled dynamics. Generalized Hamiltonian forms are exhibited and described by their series expansions in the sampling period. On these bases, higher-order approximate structure-preserving integrators are proposed. The solutions are constructive. Numerical examples illustrate the theoretical results in Section 5.

### Notations

R and N denote the set of real and natural numbers including 0, respectively. For any vector $x\in R^{n}$, |x| denotes the standard Euclidean norm and $x^{⊤}$ its transpose. Given a square matrix R≥0 and $x\in R^{n}$, the weighted square seminorm is defined as ${∥x∥}_{R}^{2}:=x^{⊤}Rx$. For *x*, $y\in R^{n}$, 〈x,y〉 denotes the inner product, i.e., $\langle x,y\rangle =x^{⊤}y$. $I_{d}$ denotes the identity function while *I* denotes the identity operator or identity matrix. Given a real-valued function $V:R^{n}\to R$ assumed differentiable, ∇V represents its gradient vector when ∇ denotes the $R^{n}$ vector of partial derivatives. Given a smooth vector field over $R^{n}$, the Lie operator is defined as $L_{f}=\sum_{i=1}^{n}f_{i}\left(x\right)\frac{\partial }{\partial x_{i}}$. ⊗ denotes the usual tensor product of matrices. A function $R\left(x,\delta \right)=O\left(\delta^{p}\right)$ is quoted in $O(\delta^{p})$, p≥1 if whenever it is defined, it can be written as $R\left(x,\delta \right)=\delta^{p-1}\tilde{R}\left(x,\delta \right)$ and there exists a function $\theta \in K_{\infty }$ and $\delta^{⋆}>0$ such that $\forall \delta \leq \delta^{⋆}$, $|\tilde{R}\left(x,\delta \right)|\leq \theta \left(\delta \right)$.

## 2. Algebraic Tools

### 2.1. The Discrete-Gradient Function

The notion of discrete gradient has been extensively studied in the literature (see [10,13,14,15] and references therein), with particular emphasis on its numerical properties and computational aspects. In what follows, we adopt the standard definition.

**Definition** **1.** *Let $H:R^{n}\to R$ be a smooth real-valued function. A discrete gradient of H is a vector-valued map denoted by $\bar{\nabla }H:R^{n}\times R^{n}\to R^{n}$, such that for all $\left(x,y\right)\in R^{n}\times R^{n}$*

(1) $$\begin{array}{cc} H(y)-H(x) & ={\bar{\nabla }}^{⊤}H\left(x,y\right)\left(y-x\right) \end{array}$$

(2) $$\begin{array}{cc} \bar{\nabla }H\left(x,x\right) & =\nabla H(x). \end{array}$$

To emphasize the variational meaning of (1), it can be rewritten in terms of the increment ϵ=y−x, leading to the equivalent algebraic identity

(3) $$\begin{array}{c} H\left(x+\epsilon \right)-H\left(x\right)-{\bar{\nabla }}^{⊤}H\left(x,x+\epsilon \right)\epsilon =0. \end{array}$$

This formulation will be useful in the sequel when discussing approximation schemes.

**Remark** **1.** *Relationship *(3)* suggests interpreting the discrete gradient as a discrete derivative of the function H at x. The existence of the discrete gradient ensures a notion of discrete differentiability (see [6,10]).*

Several elementary properties follow directly from Definition 1.

- The discrete gradient of a given function *H* is not uniquely defined.
- For any pair (x,y), differing only in the *i*-th component (i.e., an elementary variation), $y=x+e_{i}$ with $e_{i}={\left(0,\ldots ,0,y_{i}-x_{i},0,\ldots ,0\right)}^{⊤}={\left(0,\ldots ,0,e_{i},0,\ldots ,0\right)}^{⊤}$, the discrete gradient can be computed according to
  (4) $$\begin{array}{c} \bar{\nabla }H\left(x,x+e_{i}\right)={\left(0,\ldots ,\frac{H\left(x+e_{i}\right)-H\left(x\right)}{e_{i}},\ldots ,0\right)}^{⊤}. \end{array}$$
- For any $z\in R^{n}$, the identity H(y)−H(x)=H(y)−H(z)+H(z)−H(x) implies the splitting property
  $$\begin{array}{c} {\bar{\nabla }}^{⊤}H\left(x,y\right)\left(y-x\right)={\bar{\nabla }}^{⊤}H\left(z,y\right)\left(y-z\right)+{\bar{\nabla }}^{⊤}H\left(x,z\right)\left(z-x\right). \end{array}$$

It follows that different discrete gradients of the same function can be constructed by splitting the path from *x* to *y* into intermediate ones.

**Example** **1.** *Setting*

(5) $$\begin{array}{c} H\left(x\right)=x_{1}x_{2}=\frac{1}{2}x^{⊤}\left(\begin{array}{cc} 0 & 1\\ 1 & 0 \end{array}\right)x \end{array}$$

*one computes*

$${\bar{\nabla }}^{1}H\left(x,y\right)=\left(\begin{array}{c} x_{2}\\ y_{1} \end{array}\right)\;along\;the\;path\;\left(x_{1},x_{2}\right)\to \left(y_{1},x_{2}\right)\to \left(y_{1},y_{2}\right)\;and$$

$${\bar{\nabla }}^{2}H\left(x,y\right)=\left(\begin{array}{c} y_{2}\\ x_{1} \end{array}\right)\;along\;the\;path\;\left(x_{1},x_{2}\right)\to \left(x_{1},y_{2}\right)\to \left(y_{1},y_{2}\right).$$

Several formulae for computing discrete gradients have been proposed in the literature. Among them, we recall:

- the Itoh–Abe discrete gradient, also known as the coordinate increment gradient [13], defined component-wise as
  (6) $$\begin{array}{c} {\bar{\nabla }}_{IA}H\left(x,y\right)=\left(\begin{array}{c} \frac{H\left(y_{1},x_{2},\ldots ,x_{n}\right)-H\left(x\right)}{y_{1}-x_{1}}\\ \frac{H\left(y_{1},y_{2},x_{3}\ldots ,x_{n}\right)-H\left(y_{1},x_{2},\ldots ,x_{n}\right)}{y_{2}-x_{2}}\\ \vdots \\ \frac{H\left(y\right)-H\left(y_{1},\ldots ,y_{n-1},x_{n}\right)}{y_{n}-x_{n}} \end{array}\right); \end{array}$$
- the Gonzales discrete gradient [10]
  (7) $$\begin{array}{c} {\bar{\nabla }}_{G}H\left(x,y\right)=\nabla H\left(\frac{x+y}{2}\right)+\frac{\nabla H\left(y\right)-\nabla H\left(x\right)-\nabla^{⊤}H\left(\frac{x+y}{2}\right)\cdot \left(y-x\right)}{{∥y-x∥}^{2}}\left(y-x\right); \end{array}$$
- the mean-value discrete gradient [9]
  (8) $$\begin{array}{c} {\bar{\nabla }}_{MV}H\left(x,y\right)=\int_{0}^{1}\nabla H\left(x+\alpha \left(y-x\right)\right)d\alpha . \end{array}$$

**Remark** **2.** *For a quadratic function $H\left(x\right)=\frac{1}{2}x^{⊤}Px$, the mean-value discrete gradient reduces to ${\bar{\nabla }}_{MV}H\left(x,y\right)=\frac{1}{2}P\left(x+y\right)$.*

**Example** **2.** *Considering again the quadratic Hamiltonian (5), one exactly computes*

(9) $$\begin{array}{c} {\bar{\nabla }}_{MV}H\left(x,y\right)=\frac{1}{2}\left(\begin{array}{cc} 0 & 1\\ 1 & 0 \end{array}\right)\left(x+y\right)=\frac{1}{2}\left(\begin{array}{c} x_{2}+y_{2}\\ x_{1}+y_{1} \end{array}\right), \end{array}$$

*that coincides with the average of the two discrete gradients computed above*

(10) $$\begin{array}{c} {\bar{\nabla }}_{MV}H\left(x,y\right)=\frac{1}{2}\left({\bar{\nabla }}^{1}H\left(x,y\right)+{\bar{\nabla }}^{2}H\left(x,y\right)\right). \end{array}$$

Generalizing Definition 1, the notions of discrete Jacobian or discrete Hessian matrices can now be introduced.

**Definition** **2.** *Given a smooth map $T:R^{n}\to R^{n}$, we denote by ${\bar{J}}_{ac}T\left(x,y\right)$, the square matrix that satisfies the variational equality*

(11) $$\begin{array}{cc} T(y)-T(x) & ={\bar{J}}_{ac}T\left(x,y\right)\left(y-x\right) \end{array}$$

*with ${\bar{J}}_{ac}T\left(x,x\right)=J_{ac}T\left(x\right)$. Setting T(x)=∇H(x), one denotes by ${\bar{H}}_{ess}\left(x\right)={\bar{J}}_{ac}\nabla H\left(x\right)$, the discrete Hessian matrix associated with H(x) satisfying*

(12) $$\begin{array}{cc} \nabla H(y)-\nabla H(x) & ={\bar{H}}_{ess}\left(x,y\right)\left(y-x\right) \end{array}$$

*with ${\bar{H}}_{ess}\left(x,x\right)=H_{ess}\left(x\right)$, the usual Hessian matrix associated with H.*

According to the mean-value computational method (8), one gets also

(13) $$\begin{array}{cc} {\bar{H}}_{ess}\left(x,y\right) & =\int_{0}^{1}H_{ess}\left(x+\alpha \left(y-x\right)\right)d\alpha . \end{array}$$

When setting ϵ=y−x, one rewrites (12) as a nonlinear algebraic equality in ϵ to be satisfied at *x*

$$\begin{array}{c} \nabla H\left(x+\epsilon \right)-\nabla H\left(x\right)-{\bar{H}}_{ess}\left(x,x+\epsilon \right)\epsilon =0. \end{array}$$

**Remark** **3.** *It is straightforward to verify that the discrete Hessian of a quadratic function coincides with its Hessian matrix P.*

Some basic properties are easily deduced from these definitions.

**Proposition** **1.** *Given $H:R^{n}\to R$, one verifies that,*

- *(i) any convex combination of discrete gradients of H*
$$\begin{array}{c} \sum_{i}\alpha_{i}{\bar{\nabla }}^{i}H\left(x,y\right)\;with\;0<\alpha_{i}<1;\;\sum_{i}\alpha_{i}=1 \end{array}$$
  *is again a discrete gradient of H;*
- *(ii) through permutation of the arguments x and y into any discrete gradient $\bar{\nabla }H\left(x,y\right)$, one recovers a discrete gradient of H*
$$\begin{array}{c} H\left(y\right)-H\left(x\right)={\bar{\nabla }}^{⊤}H\left(y,x\right)\left(y-x\right)=-{\bar{\nabla }}^{⊤}H\left(x,y\right)\left(x-y\right). \end{array}$$

*The same properties hold for the discrete Hessian matrix (11).*

From now on, to further analyze properties of the discrete gradient or discrete Hessian described according to the mean-value method, we use, for simplicity, a notation frequently adopted in the literature setting, ${\bar{\nabla }}_{MV}H\left(x,y\right)=G\left(x,y\right)$, for a generic *H*-function. The discrete Hessian matrix, denoted by ${\bar{H}}_{ess}\left(x,y\right)$, always refers to that described by (13).

**Proposition** **2.** *Given $H:R^{n}\to R$ assumed smooth, then,*

- *(i) its mean-value discrete gradient (resp. its mean-value discrete Hessian) is invariant under permutation of its entries x and y*
$$\begin{array}{cc} G(x,y) & =G\left(y,x\right)\;and\;{\bar{H}}_{ess}\left(x,y\right)={\bar{H}}_{ess}\left(y,x\right); \end{array}$$
- *(ii) denoting by $G_{x}\left(x,y\right)$ (resp. $G_{y}\left(x,y\right)$), the partial derivative of G(x,y) with respect to x(resp. y), they are square symmetric matrices in $R^{n}\times R^{n}$ with entries $\left(x,y\right)\in R^{2n}$*
$$G_{x}^{⊤}\left(x,y\right)=G_{x}\left(x,y\right)\;and\;G_{y}^{⊤}\left(x,y\right)=G_{y}\left(x,y\right);$$
- *(iii) $G_{x}\left(x,y\right)$ and $G_{y}\left(x,y\right)$ coincide with the permutation of their entries x and y*
$$\begin{array}{c} G_{x}\left(x,y\right)=G_{y}\left(y,x\right)\;and\;G_{y}\left(x,y\right)=G_{x}\left(y,x\right); \end{array}$$
- *(iv) the mean value discrete Hessian is symmetric ${\bar{H}}_{ess}^{⊤}\left(x,y\right)={\bar{H}}_{ess}\left(x,y\right)$ and satisfies*
$$\begin{array}{c} {\bar{H}}_{ess}\left(x,y\right)=G_{x}\left(x,y\right)+G_{y}\left(x,y\right). \end{array}$$

**Proof.**  As for (i), setting α=1−β into (8), one gets

$$\begin{array}{c} G\left(x,y\right)=-\int_{1}^{0}\nabla H\left(x+\left(1-\beta \right)\left(y-x\right)\right)d\beta =\int_{0}^{1}\nabla H\left(y+\beta \left(x-y\right)\right)d\beta =G\left(y,x\right) \end{array}$$

and identically for ${\bar{H}}_{ess}\left(x,y\right)$. Points (ii) and (iii) are an immediate consequence of Proposition 1 and symmetry (i) of the mean-value gradient. As far as (iv) is concerned, one directly computes through differentiation with respect to *x* and *y* of (8)

$$\begin{array}{cc} G_{x}\left(x,y\right)+G_{y}\left(x,y\right) & =\int_{0}^{1}H_{ess}\left(x+\alpha \left(y-x\right)\right)\left(1-\alpha \right)d\alpha +\int_{0}^{1}H_{ess}\left(x+\alpha \left(y-x\right)\right)\alpha d\alpha \\ & =\int_{0}^{1}H_{ess}\left(x+\alpha \left(y-x\right)\right)d\alpha ={\bar{H}}_{ess}\left(x,y\right). \end{array}$$

□

**Example** **3.** *Setting*

$$H\left(x\right)=x_{1}x_{2}^{2}$$

*one first verifies the properties stated in Proposition 1 for a non-specific discrete gradient. In particular,*

$$\begin{array}{c} {\bar{\nabla }}^{1}H\left(x,y\right)=\left(\begin{array}{c} y_{2}^{2}\\ x_{1}\left(x_{2}+y_{2}\right) \end{array}\right)\;and\;{\bar{\nabla }}^{2}H\left(x,y\right)=\left(\begin{array}{c} x_{2}^{2}\\ y_{1}\left(y_{2}+x_{2}\right) \end{array}\right) \end{array}$$

*are two discrete-gradient vectors, each one being deduced from the other one through permutation of its entries; i.e.,*

$$\begin{array}{c} {\bar{\nabla }}^{i}H\left(y,x\right)={\bar{\nabla }}^{j}H\left(x,y\right)\;\mathrm{for}\;i\neq j,\;i,j=1,2. \end{array}$$

*Computing their partial derivatives with respect to x and y, respectively, one gets*

$$\begin{array}{c} {\bar{\nabla }}_{x}^{1}H\left(x,y\right)=\left(\begin{array}{cc} 0 & 0\\ x_{2}+y_{2} & x_{1} \end{array}\right)\;\mathrm{and}\;{\bar{\nabla }}_{y}^{1}H\left(x,y\right)=\left(\begin{array}{cc} 0 & 2y_{2}\\ 0 & x_{1}, \end{array}\right) \end{array}$$

$$\begin{array}{c} {\bar{\nabla }}_{x}^{2}H\left(x,y\right)=\left(\begin{array}{cc} 0 & 2x_{2}\\ 0 & y_{1} \end{array}\right)\;\mathrm{and}\;{\bar{\nabla }}_{y}^{2}H\left(x,y\right)=\left(\begin{array}{cc} 0 & 0\\ y_{2}+x_{2} & y_{1} \end{array}\right) \end{array}$$

*so verifying that symmetry fails but they coincide up to permutation of their entries*

$$\begin{array}{c} {\bar{\nabla }}_{x}^{i}H\left(x,y\right)={\bar{\nabla }}_{y}^{j}H\left(y,x\right)\;\mathrm{for}\;i\neq j,\;i,j=1,2. \end{array}$$

*However, their average is again a discrete gradient*

$$\begin{array}{c} \frac{{\bar{\nabla }}^{1}H\left(x,y\right)+{\bar{\nabla }}^{2}H\left(x,y\right)}{2}=\frac{1}{2}\left(\begin{array}{c} y_{2}^{2}+x_{2}^{2}\\ x_{1}x_{2}+x_{1}y_{2}+y_{1}x_{2}+y_{1}y_{2}) \end{array}\right)={\bar{\nabla }}^{3}H\left(x,y\right) \end{array}$$

*that satisfies ${\bar{\nabla }}^{3}H\left(x,y\right)={\bar{\nabla }}^{3}H\left(y,x\right)$. The partial derivatives with respect to x and y given by*

$$\begin{array}{c} {\bar{\nabla }}_{x}^{3}\left(x,y\right)=\left(\begin{array}{cc} 0 & x_{2}\\ \frac{x_{2}+y_{2}}{2} & \frac{x_{1}+y_{1}}{2} \end{array}\right)\;\mathrm{and}\;{\bar{\nabla }}_{y}^{3}\left(x,y\right)=\left(\begin{array}{cc} 0 & y_{2}\\ \frac{x_{2}+y_{2}}{2} & \frac{x_{1}+y_{1}}{2} \end{array}\right) \end{array}$$

*satisfy ${\bar{\nabla }}_{x}^{3}H\left(x,y\right)={\bar{\nabla }}_{y}^{3}H\left(y,x\right)$. This illustrates the fact that the discrete gradient is not uniquely defined and has its own symmetry properties.*

Computing the mean-value discrete gradient and discrete Hessian according to (8) and (13), one gets

$$\begin{array}{c} G\left(x,y\right)=\int_{0}^{1}\left(\begin{array}{c} {\left(x_{2}+\alpha \left(y_{2}-x_{2}\right)\right)}^{2}\\ 2(x_{1}+\alpha \left(y_{1}-x_{1}\right)\left(x_{2}+\alpha \left(y_{2}-x_{2}\right)\right) \end{array}\right)d\alpha =\frac{1}{3}\left(\begin{array}{c} x_{2}^{2}+x_{2}y_{2}+y_{2}^{2}\\ 2x_{1}x_{2}+x_{1}y_{2}+y_{1}x_{2}+2y_{1}y_{2} \end{array}\right) \end{array}$$

and

$$\begin{array}{c} {\bar{H}}_{ess}\left(x,y\right)=2\int_{0}^{1}\left(\begin{array}{cc} 0 & x_{2}+\alpha \left(y_{2}-x_{2}\right)\\ x_{2}+\alpha \left(y_{2}-x_{2}\right) & x_{1}+\alpha \left(y_{1}-x_{1}\right) \end{array}\right)d\alpha =\left(\begin{array}{cc} 0 & x_{2}+y_{2}\\ x_{2}+y_{2} & x_{1}+y_{1} \end{array}\right) \end{array}$$

so verifying the properties stated in Proposition 2; i.e.,

$$\begin{array}{c} G_{x}\left(x,y\right)\left(\begin{array}{cc} 0 & \frac{2x_{2}+y_{2}}{3}\\ \frac{2x_{2}+y_{2}}{3} & \frac{2x_{1}+y_{1}}{3} \end{array}\right)\;\mathrm{and}\;G_{y}\left(x,y\right)=\left(\begin{array}{cc} 0 & \frac{2y_{2}+x_{2}}{3}\\ \frac{x_{2}+2y_{2}}{3} & \frac{x_{1}+2y_{1}}{3} \end{array}\right) \end{array}$$

are symmetric matrices and each one recovers the other one through permutation of its entries. In addition,

$$\begin{array}{cc} {\bar{H}}_{ess} & =\left(\begin{array}{cc} 0 & \frac{2x_{2}+y_{2}}{3}\\ \frac{2x_{2}+y_{2}}{3} & \frac{2x_{1}+y_{1}}{3} \end{array}\right)+\left(\begin{array}{cc} 0 & \frac{2y_{2}+x_{2}}{3}\\ \frac{x_{2}+2y_{2}}{3} & \frac{x_{1}+2y_{1}}{3} \end{array}\right)=\left(\begin{array}{cc} 0 & x_{2}+y_{2}\\ x_{2}+y_{2} & x_{1}+y_{1} \end{array}\right)\\ & =G_{x}\left(x,y\right)+G_{y}\left(x,y\right) \end{array}$$

is a symmetric matrix that is also invariant through permutation of its entries.

### 2.2. Tensorial Exponential Representation

In the scalar case n=1, the discrete gradient that represents the variation of the *H*-function between two points can be described through the Taylor expansion of the *H*-function around *x* with respect to the increment y−x. One writes

$$H\left(y\right)=H\left(x\right)+\sum_{i\geq 1}\frac{{\left(y-x\right)}^{i}}{i!}\frac{d^{i}H\left(x\right)}{dx^{i}}$$

so getting a series expansion of the discrete-gradient vector in terms of the increment y−x

$$\bar{\nabla }H\left(x,y\right)=\nabla H\left(x\right)+\left(y-x\right)\left(\frac{1}{2}\nabla^{2}H\left(x\right)+\ldots +\frac{1}{i!}{\left(y-x\right)}^{i-2}\nabla^{i}H\left(x\right)+\ldots \right)$$

with $\nabla^{i}H\left(x\right)=\frac{d^{i}H\left(x\right)}{dx^{i}}$.

In the sequel, we generalize this representation to the *n*-dimensional case thanks to formal operators introduced in [27] to describe the evolutions of discrete-time dynamics along time. One recalls the definitions below.

**Definition** **3.** *Given a pair $\left(x,y\right)\in R^{n}\times R^{n}$ and indicating by $L_{y-x}$ the usual Lie derivative along y−x, one defines, where |y−x| are sufficiently small, through their series expansions:*

- *the tensorial exponential operator*
(14) $$\begin{array}{c} exp_{⊗L_{y-x}}:=I+L_{y-x}+\sum_{i\geq 2}\frac{1}{i!}L_{y-x}^{⊗i}; \end{array}$$
- *the discrete directional derivative*
(15) $$\begin{array}{cc} {\bar{L}}_{y-x} & :=exp_{⊗L_{y-x}}-I=\left({\left(y-x\right)}^{⊤},\ldots ,{\left({\left(y-x\right)}^{⊗i}\right)}^{⊤},\ldots \right){\left(\partial_{x},\ldots ,\frac{1}{i!}\partial_{x}^{⊗i},\ldots \right)}^{⊤} \end{array}$$

*when *⊗* indicates the tensor product of matrices, I the identity operator, and $\partial_{x}=\mathrm{column}{\left(\frac{\partial }{\partial x_{j}}\right)}_{j=1,n}$.*

The result below is an immediate consequence of the so defined operators. All the manipulations of series expansions developed in the sequel are defined for an increment |y−x| sufficiently small; a constraint that can be relaxed in the case of finite expansions.

**Lemma** **1.** *Given a smooth real valued function $H:R^{n}\to R$, one verifies $\forall \left(x,y\right)\in R^{n}\times R^{n}$*

$$\begin{array}{cc} H(y) & =exp_{⊗L_{y-x}}H{|}_{x}=exp_{⊗L_{y-x}}H\left(x\right)=H\left(x\right)+\sum_{i\geq 1}\frac{1}{i!}L_{y-x}^{⊗i}H\left(x\right). \end{array}$$

Accordingly, the discrete gradient can be represented as a differential operator of infinite order. One gets this by construction.

**Proposition** **3.** *Given a smooth real-valued function $H:R^{n}\to R$, one verifies for $\forall \left(x,y\right)\in R^{n}\times R^{n}$*

(16) $$\begin{array}{cc} H(y)-H(x) & ={\bar{L}}_{y-x}H\left(x\right)={\left(y-x\right)}^{⊤}\bar{\nabla }H\left(x,y\right) \end{array}$$

(17) $$\begin{array}{cc} \mathrm{resp}.\;\nabla H(y)-\nabla H(x) & ={\bar{L}}_{y-x}\nabla H\left(x\right)={\left(y-x\right)}^{⊤}{\bar{H}}_{ess}\left(x,y\right) \end{array}$$

*that can be interpreted as the discrete directional derivative of the H-function (resp. of ∇H(x)) along the variation y−x.*

**Proof.**  By definition of the tensorial operator and directional derivative, one has

$$\begin{array}{cc} {\bar{L}}_{y-x}H\left(x\right) & =L_{y-x}H\left(x\right)+\sum_{i\geq 2}\frac{1}{i!}L_{y-x}^{⊗i}H\left(x\right)\\ \; & ={\left(y-x\right)}^{⊤}\nabla H\left(x\right)+{\left(y-x\right)}^{⊤}\sum_{i\geq 1}\frac{1}{(i+1)!}L_{y-x}^{⊗i}\nabla H\left(x\right) \end{array}$$

noting that for i≥1, $L_{y-x}^{⊗i}H\left(x\right)={\left(y-x\right)}^{⊤}L_{y-x}^{⊗i-1}\nabla H\left(x\right)$. □

The results in Proposition 3 can be exploited to characterize the mean-value discrete gradient and discrete Hessian through their series expansions in terms of differential operators.

**Proposition** **4.** *Under the assumptions in Proposition 3:*

- *the mean-value discrete gradient (8) admits the series expansion*
  (18) $$\begin{array}{cc} G(x,y) & =\int_{0}^{1}\nabla H\left(x+\alpha \left(y-x\right)\right)d\alpha =\int_{0}^{1}exp_{⊗\alpha L_{y-x}}\nabla H\left(x\right)d\alpha \\ & =\sum_{i\geq 0}\frac{1}{(i+1)!}L_{y-x}^{⊗i}\nabla H\left(x\right) \end{array}$$
  (19) $$\begin{array}{cc} \;\;\;\;\;\;\;\;\;\;\;=\nabla H\left(x\right)+\frac{1}{2}L_{y-x}\nabla H\left(x\right)+\frac{1}{3!}L_{y-x}⊗L_{y-x}\nabla H\left(x\right)+\frac{1}{4!}L_{y-x}^{⊗3}\nabla H\left(x\right)+\ldots & \\ \;\;\;\;\;\;\;\;\;\;\;=\nabla H(x)+S(x,y-x,H)(y-x) & \end{array}$$
  *with symmetric square matrix $S\left(x,y-x,H\right)=S^{⊤}\left(x,y-x,H\right)$ given by*
  $$\begin{array}{cc} S(x,y-x,H) & =\int_{0}^{1}\alpha exp_{⊗\alpha L_{y-x}}H_{ess}\left(x\right)d\alpha =\sum_{i\geq 0}\frac{1}{(i+2)!}L_{y-x}^{⊗i}H_{ess}\left(x\right)\\ & =\frac{1}{2}H_{ess}\left(x\right)+\frac{1}{3!}L_{y-x}H_{ess}\left(x\right)+\frac{1}{4!}L_{y-x}⊗L_{y-x}H_{ess}\left(x\right)+\ldots . \end{array}$$
- *For the mean-value discrete Hessian matrix, one gets analogously*
  (20) $$\begin{array}{cc} \;{\bar{H}}_{ess}\left(x,y\right) & =\int_{0}^{1}exp_{⊗\alpha L_{y-x}}H_{ess}\left(x\right)d\alpha =\sum_{i\geq 0}\frac{1}{(i+1)!}L_{y-x}^{⊗i}H_{ess}\left(x\right)\\ & =H_{ess}\left(x\right)+\frac{1}{2}L_{y-x}H_{ess}\left(x\right)+\frac{1}{3!}L_{y-x}⊗L_{y-x}H_{ess}\left(x\right)+\ldots . \end{array}$$

**Proof.**  The proof is an immediate consequence of the definitions of the tensorial exponential and the mean-value gradient vector that give, by construction,

$$\begin{array}{cc} G(x,y) & =\int_{0}^{1}exp_{⊗\alpha L_{y-x}}\nabla H\left(x\right)d\alpha \\ & =\int_{0}^{1}\sum_{i\geq 0}\frac{\alpha^{i}}{i!}L_{y-x}^{⊗i}\nabla H\left(x\right)d\alpha =\sum_{i\geq 0}\frac{1}{(i+1)!}L_{y-x}^{⊗i}\nabla H\left(x\right). \end{array}$$

As far as the matrix S(x,(y−x),H) and the Hessian matrix are concerned, we note that, by construction, $L_{y-x}\nabla H\left(x\right)=H_{ess}\left(x\right)\left(y-x\right)$ and, more generally, $L_{y-x}⊗L_{y-x}\nabla H\left(x\right)=L_{y-x}H_{ess}\left(x\right)\left(y-x\right)\;\mathrm{up}\;\mathrm{to}\;L_{y-x}^{⊗(p+1)}\nabla H\left(x\right)=L_{y-x}^{⊗p}H_{ess}\left(x\right)\left(y-x\right),\;\forall p\geq 1$, so getting the result. Symmetry follows from the fact that each term $L_{y-x}^{⊗i}H_{ess}\left(x\right)$ is a symmetric matrix, since $H_{ess}\left(x\right)$ is a symmetric matrix. □

**Remark** **4.** *These representations induce computational facilities and give sense to approximations of increasing order with respect to y−x=ϵ of the Hamiltonian function. When the Hamiltonian is a polynomial of finite order p, these expansions are of finite order p. In more detail, adopting the variational representation at x with respect to ϵ, one gets*

$$\begin{array}{cc} G(x,x+\epsilon ) & =\nabla H\left(x\right)+\frac{1}{2}L_{\epsilon }\nabla H\left(x\right)+\frac{1}{3!}L_{\epsilon }^{⊗2}\nabla H\left(x\right)+\frac{1}{4!}L_{\epsilon }^{⊗3}\nabla H\left(x\right)+{O(|\epsilon |}^{4})\\ {\bar{H}}_{ess}\left(x,x+\epsilon \right) & =H_{ess}\left(x\right)+\frac{1}{2}L_{\epsilon }H_{ess}\left(x\right)+\frac{1}{3!}L_{\epsilon }^{⊗2}H_{ess}\left(x\right)+\frac{1}{4!}L_{\epsilon }^{⊗3}H_{ess}\left(x\right)+{O(|\epsilon |}^{4}). \end{array}$$

The next proposition characterizes the first-order approximations in the increment of the mean-value discrete gradient and discrete Hessian matrix.

**Proposition** **5.** *Under the assumptions of Proposition 3, the mean-value discrete gradient and discrete Hessian admit the first-order approximations below setting |y−x|=|ϵ| that recovers the midpoint integrator*

$$\begin{array}{cc} G_{ap}\left(x,y\right) & =\nabla H\left(\frac{x+y}{2}\right)+{O(|\epsilon |}^{2})\\ {\bar{H}}_{ess-ap}\left(x,y\right) & =H_{ess}\left(\frac{x+y}{2}\right)+{O(|\epsilon |}^{2}). \end{array}$$

**Proof.**  From Proposition 4, one easily verifies that

$$\begin{array}{cc} G(x,y) & =\nabla H\left(x\right)+\frac{1}{2}L_{y-x}\nabla H\left(x\right)+{O(|\epsilon |}^{2})=\nabla H\left(x\right)+H_{ess}\left(x\right)\frac{y-x}{2}+{O(|\epsilon |}^{2})\\ \; & =\nabla H\left(x+\frac{\left(y-x\right)}{2}\right)+{O(|\epsilon |}^{2})=\nabla H\left(\frac{x+y}{2}\right)+{O(|\epsilon |}^{2}). \end{array}$$

The same holds true for the Hessian matrix. □

**Remark** **5.** *For a quadratic function, $H\left(x\right)=\frac{1}{2}x^{⊤}Px$, one computes exactly*

$$G\left(x,y\right)=Px+\frac{1}{2}P\left(y-x\right)=\frac{1}{2}P\left(x+y\right)=G_{ap}\left(x,y\right);\;{\bar{H}}_{ess}=P.$$

**Example** **4.** *Setting again $H\left(x\right)=x_{1}x_{2}^{2}$, one computes exactly*

$$\begin{array}{ccc} G(x,y) & =\frac{1}{3}\left(\begin{array}{c} x_{2}^{2}+x_{2}y_{2}+y_{2}^{2}\\ 2x_{1}x_{2}+x_{2}y_{1}+x_{1}y_{2}+2y_{1}y_{2} \end{array}\right)\;\mathrm{and}\;{\bar{H}}_{ess}\left(x,y\right) & =\left(\begin{array}{cc} 0 & x_{2}+y_{2}\\ x_{2}+y_{2} & x_{1}+y_{1} \end{array}\right) \end{array}$$

*while the midpoint approximation scheme gives*

$$\begin{array}{ccc} G_{ap}\left(x,y\right) & =\left(\begin{array}{c} \frac{1}{2}{\left(x_{2}+y_{2}\right)}^{2}\\ \left(x_{1}+y_{1}\right)\left(x_{2}+y_{2}\right) \end{array}\right)\;\mathrm{and}\;{\bar{H}}_{ess-ap}\left(x,y\right) & =\left(\begin{array}{cc} 0 & x_{2}+y_{2}\\ x_{2}+y_{2} & x_{1}+y_{1} \end{array}\right)={\bar{H}}_{ess}\left(x,y\right). \end{array}$$

## 3. Canonical Discrete-Time Hamiltonian Dynamics

The algebraic apparatus developed in Section 2 is now applied to describe a class of discrete-time Hamiltonian dynamics and to discuss their fundamental conservative and symplectic structural properties. We start by recalling the definition of discrete-time dynamics.

**Definition** **4.** *Given a smooth map $F:R^{n}\to R^{n}$, discrete-time dynamics over $R^{n}$ are described by first-order-difference equations of the form*

(21) $$\begin{array}{c} x^{+}-x=F\left(x\right) \end{array}$$

*where $x^{+}$ denotes the state reached from x one step ahead.*

The variational format of the dynamics (21) naturally leads us to interpret F(x) as a discrete vector field with associated update map x+F(x).

### 3.1. Discrete-Time Hamiltonian Dynamics

We adopt the viewpoint, common in the control literature, to define Hamiltonian dynamics satisfying the preservation of a scalar function H(x). In classical Hamiltonian mechanics this corresponds to energy conservation that is ensured in continuous time by vector fields of the form J∇H(x). Following the same idea, canonical discrete-time Hamiltonian dynamics can be obtained by replacing the gradient with a discrete gradient that consistently represents a finite variation of *H*.

From now on, we consider dynamics of $R^{2n}$ and we denote by G(x,y) the discrete mean-value gradient defined in (8), constructed according to the tensorial expansion described in Proposition 4.

**Definition** **5.** *Given a smooth function $H:R^{2n}\to R$, a canonical discrete-time Hamiltonian dynamics are defined by the implicit difference equation*

(22) $$\begin{array}{cc} x^{+}-x & =JG(x,x^{+}) \end{array}$$

*where $J=\left[\begin{array}{cc} 0 & I_{n}\\ -I_{n} & 0 \end{array}\right]=-J^{⊤}$ is the canonical skew symmetric matrix. $JG(x,x^{+})$ is referred to as the canonical discrete-time Hamiltonian vector field.*

Some immediate remarks follow.

- Point (22) is implicitly defined due to the dependency of $G(x,x^{+})$ in both *x* and $x^{+}$.
- The Hamiltonian is, by construction, preserved along the dynamics; i.e.,
  $$H\left(x^{+}\right)-H\left(x\right)=G^{⊤}\left(x,x^{+}\right)JG\left(x,x^{+}\right)=0$$
  expressing conservativeness of *H*.

**Remark** **6.** *Replacing $G(x,x^{+})$ with any other discrete gradient associated with H preserves the conservative structure but generally destroys additional structural properties specific to the mean-value discrete gradient.*

### 3.2. Explicit Representation

We now briefly address the existence of an explicit representation of (22). Introducing the increment $\epsilon =x^{+}-x$, we rewrite the dynamics in variational form as

(23) $$\begin{array}{cc} H(x,\epsilon ) & =\epsilon -JG(x,x+\epsilon ) \end{array}$$

so that the discrete Hamiltonian vector field $\epsilon =F_{H}\left(x\right)$ is a solution to the nonlinear algebraic equation

(24) $$\begin{array}{c} H(x,\epsilon )=0. \end{array}$$

The result below follows.

**Proposition** **6.** *Let $H:R^{2n}\to R$ be smooth and let $x_{0}\in R^{2n}$ satisfy $\nabla H(x_{0})=0$. Assuming the matrix $I-\frac{1}{2}JH_{ess}\left(x_{0}\right)$ to be non-singular, then there exists a neighborhood $U(x_{0})$ and a function $F_{H}\left(x\right)$ defined on it such that $\forall x\in U(x_{0})$, $x^{+}-x=JG\left(x,x^{+}\right)$ solves (22) with $F_{H}\left(x_{0}\right)=0$.*

**Proof.**  The result follows from the Implicit Function Theorem applied to solve (24); i.e.,

$$\begin{array}{cc} H(x,\epsilon ) & =\epsilon -JG(x,x+\epsilon )=0\\ & =-J\nabla H\left(x\right)+\left(I-\frac{1}{2}JH_{ess}\left(x\right)\right)\epsilon -\frac{1}{3!}JL_{\epsilon }^{⊗2}\nabla JH\left(x\right)-\frac{1}{4!}L_{\epsilon }^{⊗3}J\nabla H\left(x\right)+{O(|\epsilon |}^{3}) \end{array}$$

that is satisfied at $\left(x,\epsilon \right)=\left(x_{0},0\right)$

$$H\left(x_{0},0\right)=-JG\left(x_{0},x_{0}\right)=J\nabla H\left(x_{0}\right)=0.$$

Being its Jacobian with respect to ϵ

$$\frac{\partial H(x,\epsilon )}{\partial \epsilon }{|}_{\left(x_{0},0\right)}=\left(I-\frac{1}{2}JH_{ess}\left(x_{0}\right)\right)$$

non-singular at $\left(x_{0},0\right)$ by assumption, a solution exists in the neighborhood of $\left(x_{0},0\right)$. □

A first-order explicit approximation is obtained by neglecting terms in $O(|\epsilon {|}^{2})$.

**Lemma** **2.** *Let $H:R^{2n}\to R$ be smooth. If the matrix $I-\frac{1}{2}JH_{ess}\left(x\right)$ is non-singular, then the dynamics*

(25) $$\begin{array}{c} x^{+}=x+{\left(I-\frac{1}{2}JH_{ess}\left(x\right)\right)}^{-1}J\nabla H\left(x\right) \end{array}$$

*provides a local explicit representation of (22).*

### 3.3. Symplectic Structure

Let us first recall some standard definitions.

**Definition** **6.** *A transformation $T:R^{2n}\to R^{2n}$ is said to be*

- *symplectic if ∀x*
  (26) $$\begin{array}{c} {\left(D_{x}T\left(x\right)\right)}^{⊤}JD_{x}T\left(x\right)=J; \end{array}$$
- *infinitesimally symplectic if ∀x*
  (27) $$\begin{array}{c} {\left(D_{x}T\left(x\right)\right)}^{⊤}J+JD_{x}T\left(x\right)=0. \end{array}$$

Symplectic transformations are volume-preserving since they satisfy $|det(D_{x}T\left(x\right))|=1$. The following properties are instrumental for the sequel. Lemma 3 below is proved in the Appendix A.

**Lemma** **3.**

- *Any infinitesimally symplectic matrix $L\in R^{2n}\times R^{2n}$ can be written as*
  (28) $$\begin{array}{c} L=JS \end{array}$$
  *for a suitably defined symmetric matrix S.*
- *The matrix*
  (29) $$\begin{array}{c} M={\left(I-JA\right)}^{-1}\left(I+JB\right) \end{array}$$
  *with A and B symmetric without eigenvalues in *1*, is symplectic if and only if*
  AJA=BJB.

A first step to investigate the symplectic property of (22) is to compute its Jacobian denoted by $D_{x}x^{+}$.

**Lemma** **4.** *The Jacobian of the canonical Hamiltonian discrete-time dynamics (22) is given by*

(30) $$\begin{array}{cc} D_{x}x^{+} & ={\left(I-JG_{x^{+}}\left(x,x^{+}\right)\right)}^{-1}\left(I+JG_{x}\left(x,x^{+}\right)\right) \end{array}$$

*provided non-singularity of the matrix $\left(I-JG_{x^{+}}\left(x,x^{+}\right)\right)$ is assumed.*

**Proof.**  Taking the derivative with respect to *x* of both sides of (22), one computes

$$\begin{array}{cc} D_{x}x^{+}=I+JG_{x}\left(x,x^{+}\right) & +JG_{x^{+}}\left(x,x^{+}\right)D_{x}x^{+} \end{array}$$

so getting the result provided non-singularity of the matrix $\left(I-JG_{x^{+}}\left(x,x^{+}\right)\right)$ is assumed. □

**Remark** **7.** *From Proposition 6, the Jacobian of the discrete-time Hamiltonian dynamics are well defined around any point at which an explicit representation exists: at any $x\in R^{2n}$ satisfying ∇H(x)=0 and non-singularity of the matrix $\left(I-\frac{1}{2}JH_{ess}\left(x\right)\right)$.*

**Remark** **8.** *We recall from Proposition 2 that $G_{x}\left(x,x^{+}\right)$ and $G_{x^{+}}\left(x,x^{+}\right)$ are symmetric matrices with symmetric entries and their sum recovers the discrete Hessian matrix, $\bar{H}\left(x,x^{+}\right)=G_{x}\left(x,x^{+}\right)+G_{x^{+}}\left(x,x^{+}\right)$. Moreover, from Proposition 4, the following expansions can be verified.*

$$\begin{array}{cc} G_{x}\left(x,x^{+}\right) & =\sum_{i\geq 0}\frac{1}{(i+2)!}L_{x^{+}-x}^{⊗i}H_{ess}\left(x\right)\\ & =\frac{1}{2}H_{ess}\left(x\right)+\frac{1}{3!}L_{x^{+}-x}H_{ess}\left(x\right)+\frac{1}{4!}L_{x^{+}-x}⊗L_{x^{+}-x}H_{ess}\left(x\right)+\ldots \\ \; & =S(x,x^{+}-x,H) \end{array}$$

*and*

$$\begin{array}{cc} G_{x^{+}}\left(x,x^{+}\right) & =\sum_{i\geq 0}\frac{i+1}{(i+2)!}L_{x^{+}-x}^{⊗i}H_{ess}\left(x\right)\\ & =\frac{1}{2}H_{ess}\left(x\right)+\frac{2}{3!}L_{x^{+}-x}H_{ess}\left(x\right)+\frac{3}{4!}L_{x^{+}-x}⊗L_{x^{+}-x}H_{ess}\left(x\right)+\ldots . \end{array}$$

*Those expansions are composed with the same terms expressing the successive derivatives of the Hessian matrix of H(x) with respect to the increment $\left(x^{+}-x\right)$ with different coefficients. They are of finite length when H(x) is a polynomial and they are equal to $\frac{1}{2}H_{ess}\left(x\right)$ when H(x) is a quadratic function.*

Necessary and sufficient conditions of symplecticity can now be stated.

**Theorem** **1.** *Given a smooth function $H:R^{2n}\to R$, then the discrete dynamics (22) is an Hamiltonian dynamics, conservative and symplectic, if and only if for all (x,y)*

(31) $$\begin{array}{c} G_{x}\left(x,y\right)JG_{x}\left(x,y\right)=G_{y}\left(x,y\right)JG_{y}\left(x,y\right). \end{array}$$

**Proof.**  From Lemma 4, the Jacobian with respect to *x* of the discrete-time dynamics has the form of *M* in (29). The result then follows from Lemma 3. □

**Remark** **9.** *The condition *(31)* characterizes symplecticity of the discrete dynamics *(22)* in terms of the two partial derivatives of the discrete gradient with respect to its arguments. A sufficient condition is given by $G_{x}\left(x,y\right)=G_{y}\left(x,y\right)$, ensuring equality of both sides of *(31)*. However, this condition is not necessary and should be seen as a particular case only. Surprising, this holds when the discrete gradient can be rewritten as a function of $\left(x+x^{+}\right)$, a condition that is properly satisfied by the midpoint integrator recalled in Proposition 5.*

Accordingly, the following result holds.

**Corollary** **1.** *Given a smooth Hamiltonian function $H:R^{2n}\to R$, consider the midpoint-like Hamiltonian dynamics*

(32) $$\begin{array}{c} x^{+}-x=J\nabla H\left(\frac{x+x^{+}}{2}\right). \end{array}$$

*Then it is symplectic while conservativeness is satisfied up to $O(|x^{+}-x{|}^{2})$ only. Its explicit form coincides with (25) in Lemma 2 in $O(|x^{+}-x{|}^{2})$.*

**Proof.**  Symplecticity follows, by construction, since

$$\frac{\partial \nabla H(\frac{x+x^{+}}{2})}{\partial x}=\frac{\partial \nabla H(\frac{x+x^{+}}{2})}{\partial x^{+}}.$$

However, from Proposition 5, $\nabla H(\frac{x+x^{+}}{2})$ approximates $G(x,x^{+})$ only up to the second order, so exact invariance of *H* is lost in general. □

For completeness, let us recall the next representation based on the further approximation of ∇H(x) in the dynamics (25) as $\nabla H\left(x\right)=\nabla H\left(x_{0}\right)+H_{ess}\left(x_{0}\right)\left(x-x_{0}\right)$. This is an approximation commonly used in the literature to describe linear approximation of discrete Hamiltonian dynamics.

**Corollary** **2.** *Given discrete Hamiltonian dynamics (22) under the assumptions of Proposition 6, with non-degenerate Hessian matrix at $x_{0}$, then $\forall x\in U(x_{0})$, the dynamics*

(33) $$\begin{array}{cc} x^{+} & ={\left(I-\frac{1}{2}JH_{ess}\left(x_{0}\right)\right)}^{-1}\left(I+\frac{1}{2}JH_{ess}\left(x_{0}\right)\right)x \end{array}$$

*are conservative and symplectic; they correspond to the discrete-time Hamiltonian dynamics built on the quadratic approximation of the given function H(x) around $x_{0}$.*

The above discussion clarifies the fact that both the symplectic property and conservativeness are not simultaneously satisfied in a nonlinear context in general.

**Remark** **10.** *We note that from (iii) in Proposition 2, the condition (31) rewrites*

$$G_{x}\left(x,x^{+}\right)JG_{x}\left(x,x^{+}\right)=G_{x}\left(x^{+},x\right)JG_{x}\left(x^{+},x\right)$$

*or, equivalently,*

$$G_{x^{+}}\left(x,x^{+}\right)JG_{x^{+}}\left(x,x^{+}\right)=G_{x^{+}}\left(x^{+},x\right)JG_{x^{+}}\left(x^{+},x\right).$$

**Remark** **11.** *The dynamics (22) is symplectic in $O{\left(x^{+}-x\right)}^{2}$.*

**Remark** **12.** *In the two-dimensional case (n=1), given that $G_{x}\left(x,x^{+}\right)$ is a symmetric matrix, then $G_{x}\left(x,x^{+}\right)JG_{x}\left(x,x^{+}\right)=\left|G_{x}\right|J$ when denoted by $|G_{x}|$, the determinant of $G_{x}$, so we can rewrite condition (31) as*

$$|G_{x}|\left(x,x^{+}\right)=\left|G_{x}\right|\left(x^{+},x\right).$$

### 3.4. Linear Discrete-Time Hamiltonian Dynamics

Let us now assume a quadratic Hamiltonian function

$$H\left(x\right)=\frac{1}{2}x^{⊤}Px,$$

where $P\in R^{2n\times 2n}$ is symmetric and positive definite. In this case, the results discussed above can be made explicit.

- According to Proposition 4 and formulae (8), the mean-value discrete gradient can be expressed explicitly as a function of the sum $x+x^{+}$:
  (34) $$\begin{array}{cc} \bar{\nabla }H\left(x,x^{+}\right) & =\int_{0}^{1}\nabla H(x+\alpha \left(y-x\right)d\alpha =\nabla H\left(x\right)+\frac{1}{2}H_{ess}\left(x^{+}-x\right)\\ \; & =Px+\frac{1}{2}P\left(x^{+}-x\right)=\frac{1}{2}P\left(x+x^{+}\right). \end{array}$$
- A canonical linear discrete-time Hamiltonian dynamics are, therefore, described through the implicit relation
  (35) $$\begin{array}{c} x^{+}-x=\frac{1}{2}JP\left(x+x^{+}\right). \end{array}$$
- An explicit representation exists whenever $\left(I-\frac{1}{2}JP\right)$ is invertible (ensured, for instance, if *P* is positive definite), yielding, from (35), to
  (36) $$\begin{array}{c} x^{+}={\left(I-\frac{1}{2}JP\right)}^{-1}\left(I+\frac{1}{2}JP\right)x. \end{array}$$
- Conservativeness and the symplectic property hold globally, by construction.

In this linear setting, necessary and sufficient conditions for a discrete vector field to be Hamiltonian can be fully characterized.

**Theorem** **2.** *A discrete-time dynamics $x^{+}=Ax$ on $R^{2n}$ is a canonical discrete-time Hamiltonian dynamic if and only if A is symplectic and has no eigenvalues equal to −1. In that case, it is associated with the quadratic Hamiltonian*

(37) $$\begin{array}{c} H\left(x\right)=\frac{1}{2}x^{⊤}Px=x^{⊤}J^{⊤}\left(A-I\right){\left(A+I\right)}^{-1}x. \end{array}$$

*The corresponding implicit representation is*

(38) $$\begin{array}{c} x^{+}-x=J\frac{P}{2}\left(x+x^{+}\right)=\left(A-I\right){\left(A+I\right)}^{-1}\left(x+x^{+}\right) \end{array}$$

*or, equivalently,*

$$A={\left(I-J\frac{P}{2}\right)}^{-1}\left(I+J\frac{P}{2}\right).$$

**Proof.**  From Lemma 3 with M=A, the assumption that *A* is symplectic with no eigenvalues equal to −1 is equivalent to the existence of an infinitesimally symplectic matrix *L* and a symmetric matrix *S* such that

$$L=JS,\;A={\left(I-JS\right)}^{-1}\left(I+JS\right).$$

The result follows by setting

$$P=2S=J^{⊤}\left(A-I\right){\left(A+I\right)}^{-1}$$

that also gives

$$A={\left(I-J\frac{P}{2}\right)}^{-1}\left(I+J\frac{P}{2}\right).$$

□

**Remark** **13.** *In this linear case, there is a clear equivalence between an implicit representation of the dynamics (35) defined by the infinitesimally symplectic matrix $\frac{1}{2}JP$, and an explicit representation (36) defined by the symplectic matrix ${\left(I-J\frac{P}{2}\right)}^{-1}\left(I+J\frac{P}{2}\right)$. The latter ensures volume preservation, i.e., |det(A)|=1.*

**Remark** **14.** *A symplectic matrix A has no eigenvalues equal to −1 if and only if the associated infinitesimally symplectic matrix JP has no eigenvalues equal to *2*. This condition is satisfied, in particular, when $H\left(x\right)=\frac{1}{2}x^{⊤}Px$ is positive definite.*

## 4. Hamiltonian Dynamics Under Sampling 

In this section we investigate how the properties characterizing a continuous-time Hamiltonian system are affected through exact or approximate sampling. In particular, we address the following question: does the exact sampling of a continuous-time Hamiltonian system yield a discrete-time Hamiltonian dynamics in the sense of Definition 5?

This question is not trivial. On one hand, from the previous section, the structure introduced in Definition 5 is symplectic under condition (31) in Theorem 1. On the other hand, it is well known that the exact sampled dynamics of a continuous-time Hamiltonian system preserves both conservativeness and the symplectic structure at the sampling instants. Hereinafter, as usual in the sampled-data context, the sampling period δ>0 is assumed sufficiently small.

### 4.1. The Nonlinear Case

Consider a nonlinear Hamiltonian function $H:R^{2n}\to R$ and the associated canonical continuous-time dynamics

(39) $$\begin{array}{c} \dot{x}\left(t\right)=J\nabla H\left(x\left(t\right)\right)=f_{H}\left(x\left(t\right)\right). \end{array}$$

By construction, the dynamics are conservative since

$$\begin{array}{c} \frac{d}{dt}H\left(x\left(t\right)\right)=\nabla^{⊤}H\left(x\left(t\right)\right)J\nabla H\left(x\left(t\right)\right)=0. \end{array}$$

Sampling the continuous-time dynamics with sampling period δ>0, and denoting $x_{k}$ by *x* and $x_{k+1}$ by $x^{+}$, one obtains the exact sampled dynamic

(40) $$\begin{array}{c} x^{+}=x+F_{H}^{\delta }\left(x\right)=e^{\delta f_{H}}x \end{array}$$

that matches the continuous trajectory at the sampling instants. Therefore, the sampled equivalent to (39) inherits its fundamental properties; namely, conservativeness and symplecticity. This is formally resumed below.

**Proposition** **7.** *The exact sampled dynamic (40) associated with (39) is conservative and symplectic; i.e.,*

$$H\left(x^{+}\right)-H\left(x\right)=0\;\mathrm{and}\;M^{\delta }{\left(x\right)}^{⊤}JM^{\delta }\left(x\right)=J$$

*where $M^{\delta }\left(x\right)=D_{x}\left(e^{\delta f_{H}}x\right)$ is the Jacobian of the flow $e^{\delta f_{H}}x$.*

**Proof.**  Conservativeness immediately follows from integration between two time steps of the dynamics (39); i.e.,

$$\begin{array}{cc} H\left(x^{+}\right)-H\left(x\right)=e^{\delta f_{H}}H\left(x\right)-H\left(x\right) & =\int_{0}^{\delta }\nabla^{⊤}H\left(x\left(t\right)\right)\dot{x}\left(t\right)dt\\ & =\int_{0}^{\delta }\nabla^{⊤}H\left(x\left(t\right)\right)J\nabla H\left(x\left(t\right)\right)dt=0. \end{array}$$

As for symplecticity, we show that $\frac{d}{d\delta }M^{\delta }{\left(x\right)}^{⊤}JM^{\delta }\left(x\right)=0$, which enables one to conclude that $M^{\delta }{\left(x\right)}^{⊤}JM^{\delta }\left(x\right)=M^{0}{\left(x\right)}^{⊤}JM^{0}\left(x\right)=J$. With this in mind, from the equality

$$\frac{de^{\delta J\nabla H}x}{d\delta }=J\nabla H\left(e^{\delta J\nabla H}x\right)$$

one computes

$$\begin{array}{c} \frac{dM^{\delta }\left(x\right)}{d\delta }={\dot{M}}^{\delta }\left(x\right)=JH_{ess}\left(e^{\delta f_{H}}x\right)D_{x}\left(e^{\delta f_{H}}x\right)=JH_{ess}\left(e^{\delta f_{H}}x\right)M^{\delta }\left(x\right) \end{array}$$

and, thus,

$$\begin{array}{cc} \; & {\dot{M}}^{\delta }{\left(x\right)}^{⊤}JM^{\delta }\left(x\right)+M^{\delta }{\left(x\right)}^{⊤}J{\dot{M}}^{\delta }\left(x\right)\\ \; & =M^{\delta }{\left(x\right)}^{⊤}H_{ess}\left(e^{\delta f_{H}}x\right)J^{⊤}JM^{\delta }\left(x\right)+M^{\delta }{\left(x\right)}^{⊤}J^{2}H_{ess}\left(e^{\delta f_{H}}x\right)M^{\delta }\left(x\right)=0 \end{array}$$

because $H_{ess}\left(x\right)$ is a symmetric matrix. This concludes the proof. □

To further investigate the properties of the sampled dynamics in a discrete-time Hamiltonian framework, we first relate the sampled increment $F_{H}^{\delta }\left(x\right)$ in (40) to the underlying continuous vector field $f_{H}\left(x\right)$ in (39). The results below specify the series representations of the discrete-gradient apparatus when the underlying discrete dynamics are the exact sampled flow.

**Lemma** **5.** *The increment $F_{H}^{\delta }\left(x\right)$ of the sampled dynamics associated with $f_{H}\left(x\right)$ can be written as*

$$\begin{array}{cc} x^{+}-x & =F_{H}^{\delta }\left(x\right)=\delta M^{\delta }\left(x,f_{H}\right)f_{H}\left(x\right) \end{array}$$

*where*

$$\delta M^{\delta }\left(x,f_{H}\right)=\int_{0}^{\delta }M^{\alpha }\left(x\right)d\alpha$$

*and $M^{\delta }\left(x\right)$ is the symplectic Jacobian of the flow. In more detail, one computes*

$$\begin{array}{cc} F_{H}^{\delta }\left(x\right) & =\delta \left(I+\frac{\delta }{2}JH_{ess}\left(x\right)+\frac{\delta^{2}}{3!}\left(JL_{J\nabla H}H_{ess}\left(x\right)+{\left(JH_{ess}\left(x\right)\right)}^{2}\right)\right)J\nabla H\left(x\right)+O\left(\delta^{4}\right). \end{array}$$

Further on, the relationship below holds when setting $\bar{\nabla }H\left(x,x+F_{H}^{\delta }\left(x\right)\right)=G\left(x,x+F_{H}^{\delta }\left(x\right)\right)$.

**Remark** **15.** *We note that in this sampled-data case, the existence of the flow $x+F_{H}^{\delta }\left(x\right)$, ensured for δ being sufficiently small, corresponds to the condition $|x^{+}-x|$ being sufficiently small, as required to define infinite series expansions.*

**Corollary** **3.** *The discrete gradient of H along the sampled dynamics (40) can be expressed in terms of the usual gradient as*

$$\begin{array}{cc} G(x,x+F_{H}^{\delta }\left(x\right)) & =\left(I+\delta S\left(x,F_{H}^{\delta },H\right)M^{\delta }\left(x,f_{H}\right)J\right)\nabla H\left(x\right)\\ \mathrm{with}\;\mathrm{symmetric}\;\mathrm{matrices}\;\;S(x,F_{H}^{\delta },H) & =\int_{0}^{1}\alpha {\bar{H}}_{ess}\left(x,x+\alpha F_{H}^{\delta }\left(x\right)\right)d\alpha \\ \mathrm{and}\;{\bar{H}}_{ess}\left(x,x+F_{H}^{\delta }\left(x\right)\right) & =\int_{0}^{1}H_{ess}\left(x+\alpha F_{H}^{\delta }\left(x\right)\right)d\alpha . \end{array}$$

*For the first terms of these expansions, one computes*

$$\begin{array}{cc} G(.) & =\nabla H\left(x\right)+\frac{\delta }{2}H_{ess}\left(x\right)J\nabla H\left(x\right)+\frac{\delta^{2}}{4}{\left(H_{ess}\left(x\right)J\right)}^{2}\nabla H\left(x\right)+\frac{\delta^{2}}{3!}L_{J\nabla H}H_{ess}\left(x\right)J\nabla H\left(x\right)+O\left(\delta^{3}\right)\\ S(.) & =\frac{1}{2}H_{ess}\left(x\right)+\frac{\delta }{3!}L_{J\nabla H}H_{ess}\left(x\right)+\frac{\delta^{2}}{4!}L_{J\nabla H}^{⊗2}H_{ess}\left(x\right)+\frac{\delta^{2}}{3!2}L_{JH_{ess}J\nabla H}H_{ess}\left(x\right)+O\left(\delta^{3}\right)\\ {\bar{H}}_{ess}\left(.\right) & =H_{ess}\left(x\right)+\frac{\delta }{2}L_{J\nabla H}H_{ess}\left(x\right)+\frac{\delta^{2}}{3!}L_{J\nabla H}^{⊗2}H_{ess}\left(x\right)+\frac{\delta^{2}}{4}L_{JH_{ess}J\nabla H}H_{ess}\left(x\right)+O\left(\delta^{3}\right). \end{array}$$

**Proof.**  For this, it is sufficient to rewrite according to Proposition 3

$$\begin{array}{cc} G(x,x+F_{H}^{\delta }\left(x\right)) & =\int_{0}^{1}\nabla H\left(x+\alpha F_{H}^{\delta }\left(x\right)\right)d\alpha =\nabla H\left(x\right)+S\left(x,F_{H}^{\delta },H\right)F_{H}^{\delta }\left(x\right) \end{array}$$

so getting the result when substituting from Lemma 5, $F_{H}^{\delta }\left(x\right)$ with $\delta M^{\delta }\left(x,f_{H}\right)f_{H}\left(x\right)$ and $f_{H}\left(x\right)$ with J∇H(x). □

**Remark** **16.** *For completeness, the expressions detailed in Remark 8 are specific to the sampled context. One gets*

$$\begin{array}{cc} G_{x}\left(.\right) & =S\left(.\right)=\frac{1}{2}H_{ess}\left(x\right)+\frac{\delta }{3!}L_{J\nabla H}H_{ess}\left(x\right)+\frac{\delta^{2}}{4!}L_{J\nabla H}^{⊗2}H_{ess}\left(x\right)+\frac{\delta^{2}}{3!2}L_{JH_{ess}J\nabla H}H_{ess}\left(x\right)+O\left(\delta^{3}\right)\\ G_{x^{+}}\left(.\right) & =\frac{1}{2}H_{ess}\left(x\right)+\frac{2\delta }{3!}L_{J\nabla H}H_{ess}\left(x\right)+\frac{3\delta^{2}}{4!}L_{J\nabla H}^{⊗2}H_{ess}\left(x\right)+\frac{\delta^{2}}{3!}L_{JH_{ess}J\nabla H}H_{ess}\left(x\right)+O\left(\delta^{3}\right) \end{array}$$

*so verifying that these expansions differ from their coefficients in δ and from the order one in δ. It is also immediately verified that quadraticity of the H-function implies $G_{x}=G_{x^{+}}=\frac{1}{2}H_{ess}\left(x\right)$.*

The next result recalled from [16] shows that the exact sampled dynamics admits what we call a generalized Hamiltonian representation, where the canonical interconnection matrix is replaced by a state-dependent one, defined through it expansion in δ.

**Theorem** **3.** *The exact sampled dynamics (40) associated with (39) satisfies the implicit relation*

(41) $$\begin{array}{cc} x^{+}-x & =J^{\delta }\left(x\right)G\left(x,x^{+}\right) \end{array}$$

*where $G(x,x^{+})$ is the mean-value discrete gradient of H and*

(42) $$\begin{array}{c} J^{\delta }\left(x\right)=\delta J+\sum_{p\geq 2}\delta^{p}J_{p}\left(x\right) \end{array}$$

*is the solution to the equality of series*

(43) $$\begin{array}{cc} \delta M^{\delta }\left(x,f_{H}\right)J & =J^{\delta }\left(x\right)\left(I+\delta S\left(x,F_{H}^{\delta },H\right)M^{\delta }\left(x,f_{H}\right)J\right). \end{array}$$

*For the first terms, one computes*

(44) $$\begin{array}{c} J_{1}=J,\;J_{2}=0,\;J_{3}=-\frac{1}{12}{\left(JH_{ess}\left(x\right)\right)}^{2}J. \end{array}$$

*By construction, the dynamics (41) globally preserves (i.e., for all δ≥0) both conservativeness and symplecticity.*

**Proof.**  From Lemma 5 and Corollary 3, to satisfy the required property, one has to verify the equality of series

$$\begin{array}{c} \delta M^{\delta }\left(x,f_{H}\right)J\nabla H\left(x\right)=J^{\delta }\left(x\right)\left(I+\delta S\left(x,F_{H}^{\delta },H\right)M^{\delta }\left(x,f_{H}\right)J\right)\nabla H\left(x\right). \end{array}$$

A solution is provided when setting $J^{\delta }\left(x\right)$, solution of (43), so getting also

$$\begin{array}{c} J^{\delta }\left(x\right)=\delta M^{\delta }\left(x,f_{H}\right)J{\left(I+\delta S\left(x,F_{H}^{\delta },H\right)M^{\delta }\left(x,f_{H}\right)J\right)}^{-1} \end{array}$$

which is well defined since

$$\begin{array}{c} I+\delta S\left(x,F_{H}^{\delta },H\right)M^{\delta }\left(x,f_{H}\right)J=I+\frac{\delta }{2}H_{ess}\left(x\right)J+O\left(\delta^{2}\right) \end{array}$$

is non-singular for all δ. To simplify the computations of the first terms, one substitutes the expansion (42) with $J^{\delta }\left(x\right)$ into the expression (43) and compares the terms of the same power in δ, so getting (44) for the first terms. □

Some further comments are in order.

- Even though conservativeness derives from the equality
  (45) $$\begin{array}{cc} H\left(x^{+}\right)-H\left(x\right) & =\int_{0}^{\delta }\nabla^{⊤}H\left(x\left(t\right)\right)J\nabla H\left(x\left(t\right)\right)dt={\bar{\nabla }}^{⊤}H\left(x,x^{+}\right)J^{\delta }\left(x\right)\bar{\nabla }H\left(x,x^{+}\right)=0 \end{array}$$
  this does not imply skew symmetry of the interconnection matrices $J^{\delta }\left(x\right)$.
- Skew symmetry up to $J_{3}$ is satisfied.
- The equality
  $${\left(I+J^{\delta }\left(x\right)G_{x}\right)}^{⊤}{\left(I-J^{\delta }\left(x\right)G_{x^{+}}\right)}^{-⊤}J{\left(I-J^{\delta }\left(x\right)G_{x^{+}}\right)}^{-1}\left(I+J^{\delta }\left(x\right)G_{x}\right)=J=M^{\delta }\left(x\right)JM^{\delta }\left(x\right)$$
  is guaranteed by symplecticity of the flow.
- (41) provides a generalized Hamiltonian implicit representation of the exact sampled dynamics $x^{+}=x+F_{H}^{\delta }\left(x\right)$.

**Remark** **17.** *It is interesting to investigate the existence of a modified Hamiltonian function, say $H^{\delta }\left(x\right)=\delta H+\sum_{p\geq 1}\delta^{p}H_{p}\left(x\right)$, so as to rewrite the implicit form (41) as a canonical Hamiltonian form with respect to $H^{\delta }\left(x\right)$ of the form $J\bar{\nabla }H^{\delta }\left(x,x^{+}\right)$. Solving the equality*

$$J^{\delta }\left(x\right)G\left(x,x^{+}\right)=J\bar{\nabla }H^{\delta }\left(x,x^{+}\right)$$

*rewritten according to the definition of the discrete gradient as*

$$\begin{array}{cc} \; & J^{\delta }\left(x\right)G\left(x,x^{+}\right)=J^{\delta }\left(x\right)\int_{0}^{1}\nabla H\left(x+\alpha \left(x^{+}-x\right)\right)d\alpha =\int_{0}^{1}J^{\delta }\left(x\right)\nabla H\left(x+\alpha \left(x^{+}-x\right)\right)d\alpha \\ \; & =J\int_{0}^{1}\nabla H^{\delta }\left(x+\alpha \left(x^{+}-x\right)\right)d\alpha =\int_{0}^{1}J\nabla H^{\delta }\left(x+\alpha \left(x^{+}-x\right)\right)d\alpha \end{array}$$

*it turns out that $H^{\delta }\left(x\right)$ should be the solution to the system of partial derivative equations*

(46) $$\begin{array}{c} J\nabla H^{\delta }\left(x\right)=J^{\delta }\left(x\right)\nabla H\left(x\right). \end{array}$$

*However, the existence of such $H^{\delta }\left(x\right)$ requires symmetry of its Hessian given by*

$$\begin{array}{c} H_{ess}^{\delta }\left(x\right)=J^{⊤}D_{x}\left(J^{\delta }\left(x\right)\nabla H\left(x\right)\right) \end{array}$$

*a property that is not a priori satisfied. As an example, for the first terms, one computes the solutions $H_{1}=H$, $H_{2}=0$ and $H_{3}$ solution of*

$$\begin{array}{c} \nabla H_{3}=-\frac{1}{12}H_{ess}\left(x\right)JH_{ess}\left(x\right)J\nabla H\left(x\right). \end{array}$$

*Computing the Jacobian of $\nabla H_{3}\left(x\right)$, one gets*

$$\begin{array}{cc} D_{x}\left(\nabla H_{3}\left(x\right)\right) & =-\frac{1}{12}D_{x}\left(H_{ess}\left(x\right)JH_{ess}\left(x\right)J\nabla H\left(x\right)\right)\\ & =-\frac{1}{12}H_{ess}\left(x\right)JH_{ess}\left(x\right)JH_{ess}\left(x\right)-\frac{1}{12}\frac{\partial H_{ess}\left(x\right)JH_{ess}\left(x\right)}{\partial x}J\nabla H\left(x\right) \end{array}$$

*with a second term that may be not symmetric.*

### 4.2. The Linear Case

The previous results are now specified to the linear case taking advantage of the quadraticity of the Hamiltonian function when setting $H\left(x\right)=\frac{1}{2}x^{⊤}Px$. Let

(47) $$\begin{array}{c} \dot{x}\left(t\right)=J\nabla H\left(x\left(t\right)\right)=JPx\left(t\right) \end{array}$$

that is, by construction, conservative and symplectic. Given a sampling period δ>0, let its sampled equivalent dynamics

(48) $$\begin{array}{c} x^{+}=e^{\delta JP}x=x+F^{\delta }x \end{array}$$

that preserves linearity and conservativeness since

$$\begin{array}{c} H\left(x^{+}\right)-H\left(x\right)=\int_{0}^{\delta }\dot{H}\left(x\left(t\right)\right),dt=\int_{0}^{\delta }x^{⊤}\left(t\right)PJPx\left(t\right)dt=0. \end{array}$$

Moreover, symplecticity holds, since for t=δ, the sampled flow satisfies ${\left(e^{\delta JP}\right)}^{⊤}Je^{\delta JP}=J$.

On these bases, let us first specify Theorem 3.

**Theorem** **4.** *The exact sampled equivalent (48) to the Hamiltonian dynamics (47) admits the generalized Hamiltonian implicit representation*

(49) $$\begin{array}{c} x^{+}-x=J^{\delta }\frac{P}{2}\left(x+x^{+}\right) \end{array}$$

*where the interconnection matrix $J^{\delta }$, described by its expansion*

$$\begin{array}{c} J^{\delta }=\delta \left(I+\sum_{n\geq 2}\frac{2^{2n}\left(2^{2n}-1\right)B_{2n}}{(2n)!}{\left(\frac{\delta JP}{2}\right)}^{2n-2}\right)J=\delta J+\sum_{i\geq 1}\delta^{2i+1}J_{2i+1}. \end{array}$$

*is skew-symmetric and satisfies the equality*

$$J^{\delta }\frac{P}{2}=tanh\left(\frac{\delta JP}{2}\right)$$

*with*

$$tanh\left(\frac{x}{2}\right)=\frac{e^{x}-1}{e^{x}+1}=\sum_{n\geq 1}\frac{2^{2n}\left(2^{2n}-1\right)B_{2n}}{(2n)!}{\left(\frac{x}{2}\right)}^{2n-1},\;B_{2n}\;\mathrm{Bernoulli}\;\mathrm{numbers}.$$

*The dynamics (49) is conservative with respect to H(x). Its explicit form*

(50) $$\begin{array}{c} x^{+}={\left(I-J^{\delta }\frac{P}{2}\right)}^{-1}\left(I+J^{\delta }\frac{P}{2}\right)x=e^{\delta JP}x \end{array}$$

*is, by construction, symplectic.*

**Proof.**  Skew-symmetry of $J^{\delta }$ follows, by construction: each term in the series defining $J^{\delta }$ involves an odd power of *J*, of the form ${\left(JP\right)}^{2i}J$, that is skew-symmetric since *P* is symmetric and $J^{⊤}=-J$. □

We note that in this linear case, skew symmetry of $J^{\delta }$ directly follows from its structure. Moreover, the result discussed in Remark 17 holds true, so proving the existence of a modified Hamiltonian function $H^{\delta }\left(x\right)$, with respect to which a canonical Hamiltonian form is recovered. One gets the following:

**Theorem** **5.** *The exact sampled equivalent (48) to the Hamiltonian dynamics (47) admits the discrete-time canonical linear Hamiltonian representation*

(51) $$\begin{array}{c} x^{+}-x=J\bar{\nabla }H^{\delta }\left(x,x^{+}\right)=J\frac{P^{\delta }}{2}\left(x+x^{+}\right) \end{array}$$

*with respect to the Hamiltonian function $H^{\delta }\left(x\right)=\frac{1}{2}x^{⊤}P^{\delta }x$, described by*

(52) $$\begin{array}{c} \frac{P^{\delta }}{2}=J^{⊤}\left(e^{\delta JP}-I\right){\left(e^{\delta JP}+I\right)}^{-1}=J^{⊤}tanh\left(\frac{\delta JP}{2}\right). \end{array}$$

*The implicit dynamics (51) is globally conservative for $H^{\delta }\left(x\right)$ for all δ≥0. Its explicit form*

(53) $$\begin{array}{c} x^{+}={\left(I-J\frac{P^{\delta }}{2}\right)}^{-1}\left(I+J\frac{P^{\delta }}{2}\right)x=e^{\delta JP}x \end{array}$$

*is globally symplectic, by construction.*

**Proof.**  The result follows by substituting from (48), $x^{+}$ with $e^{\delta JP}x$ into (51). Symmetry of $P^{\delta }$ follows since each term in its expansion is of the form $P{\left(JP\right)}^{2i}$, hence symmetric. □

**Corollary** **4.** *For the first terms, one computes*

$$\begin{array}{cc} P^{\delta } & =\delta P\left(I+\sum_{n\geq 2}\frac{2^{2n}\left(2^{2n}-1\right)B_{2n}}{(2n)!}{\left(\frac{\delta JP}{2}\right)}^{2n-2}\right)=\delta P-\frac{\delta^{3}}{12}PJPJP+\frac{\delta^{5}}{120}P{\left(JP\right)}^{4}+O\left(\delta^{7}\right)\\ J^{\delta } & =\delta \left(I+\sum_{n\geq 2}\frac{2^{2n}\left(2^{2n}-1\right)B_{2n}}{(2n)!}{\left(\frac{\delta JP}{2}\right)}^{2n-2}\right)J=\delta J-\frac{\delta^{3}}{12}JPJPJ+\frac{\delta^{5}}{120}{\left(JP\right)}^{4}J+O\left(\delta^{7}\right). \end{array}$$

**Remark** **18.** *We note that in this linear context, the set of PDE to be solved, described in the nonlinear context by (46), reduces to the equality of matrices*

$$\begin{array}{c} J^{\delta }\frac{P}{2}=J\frac{P^{\delta }}{2}=tanh\left(\frac{\delta JP}{2}\right). \end{array}$$

In conclusion, under exact sampling, one obtains two equivalent implicit representations of the explicit dynamics (48), either in terms of a modified Hamiltonian function $H^{\delta }\left(x\right)$ or a modified skew symmetric interconnection matrix $J^{\delta }$.

In the sequel, the function $H^{\delta }\left(x\right)$ will be referred to as the *sampled Hamiltonian*.

The following result is an immediate consequence of this equivalence.

**Corollary** **5.** *Given the linear Hamiltonian dynamics *(47)*, the function $H\left(x\right)=\frac{1}{2}x^{⊤}Px$ and the family of functions $H^{\delta }\left(x\right)=\frac{1}{2}x^{⊤}P^{\delta }x$ are constants of motion of the sampled dynamics *(48)*; i.e., ∀δ>0*

$$\begin{array}{cc} H\left(x^{+}\right)-H\left(x\right) & ={\left(x+x^{+}\right)}^{⊤}PJ^{\delta }P\left(x+x^{+}\right)=0\\ H^{\delta }\left(x^{+}\right)-H^{\delta }\left(x\right) & ={\left(x+x^{+}\right)}^{⊤}P^{\delta }JP^{\delta }\left(x+x^{+}\right)=0. \end{array}$$

**Remark** **19.** *In dimension 2 (n=1), one obtains $P^{\delta }=\delta \alpha^{\delta }\left(|P|\right)P$ and $J^{\delta }=\delta \alpha^{\delta }\left(|P|\right)J$, where*

$$\alpha^{\delta }\left(|P|\right)=1+\frac{\delta^{2}}{12}{\left|P\right|}^{2}+\frac{\delta^{4}}{120}{\left|P\right|}^{4}+\frac{17\delta^{6}}{20160}{\left|P\right|}^{6}+\ldots$$

*and |P| denotes the determinant of P. The sampled dynamics reads*

$$x^{+}-x=\delta \frac{\alpha^{\delta }\left(|P|\right)}{2}JP\left(x+x^{+}\right)$$

*with explicit form*

$$x^{+}={\left(I-\delta \frac{\alpha^{\delta }\left(|P|\right)}{2}JP\right)}^{-1}\left(I+\delta \frac{\alpha^{\delta }\left(|P|\right)}{2}JP\right)x=e^{\delta JP}x.$$

### 4.3. About Approximations of Sampled Hamiltonian Dynamics

In the sequel, we discuss different approximation methods of the exact Hamiltonian form described in (41), according to the preservation of their qualifying properties. For, we recall that a property will be said to hold in $O(\delta^{p})$ if the algebraic equality that qualifies this property is satisfied up to an error in $O(\delta^{p})$.

#### 4.3.1. Conservative Approximation

A commonly adopted representation of discrete-time Hamiltonian dynamics (see [26]) is obtained by replacing in the continuous-time one the gradient with a discrete gradient and the time-derivative of the state by the first-order increment. This leads us to define the discrete analogue to (39) as

(54) $$\begin{array}{c} x^{+}-x=\delta JG\left(x,x^{+}\right). \end{array}$$

Invariance of the Hamiltonian H(x) holds, by construction, since

$$\begin{array}{c} H\left(x^{+}\right)-H\left(x\right)=G{\left(x,x^{+}\right)}^{⊤}\left(x^{+}-x\right)=\delta G{\left(x,x^{+}\right)}^{⊤}JG\left(x,x^{+}\right)=0 \end{array}$$

by skew-symmetry of *J*. However, symplecticity is lost in general and is violated in $O(\delta^{3})$; in fact, easy computations show that

$$\begin{array}{c} {\left(I+\delta JG_{x}\right)}^{⊤}{\left(I-\delta JG_{x^{+}}\right)}^{-⊤}J{\left(I-\delta JG_{x^{+}}\right)}^{-1}\left(I+\delta JG_{x}\right), \end{array}$$

differs from *J* bythe terms in $O(\delta^{3})$. This is consistent with the fact that the expansion of the right-hand side of (54) (see Lemma 5), computed as,

$$\begin{array}{cc} \delta JG(x,x+F_{H}^{\delta }\left(x\right)) & =\delta J\nabla H\left(x\right)+\frac{\delta^{2}}{2}JH_{\mathrm{ess}}\left(x\right)J\nabla H\left(x\right)\\ \; & +\frac{\delta^{3}}{4}{\left(JH_{\mathrm{ess}}\left(x\right)\right)}^{2}J\nabla H\left(x\right)+\frac{\delta^{3}}{3!}JL_{J\nabla H}H_{\mathrm{ess}}\left(x\right)J\nabla H\left(x\right)+\ldots \end{array}$$

differs from $F_{H}^{\delta }\left(x\right)$ by the terms in $O(\delta^{3})$.

#### 4.3.2. Implicit Midpoint or Symplectic Integrator

An alternative to (54) is provided by the implicit midpoint rule or symplectic integrator (see [18]), so considering, instead of (54), the dynamics

(55) $$\begin{array}{c} x^{+}-x=\delta J\nabla H\;\left(\frac{x+x^{+}}{2}\right). \end{array}$$

In this case, the dynamics are symplectic, by construction, due to the equality of the two partial derivatives

$$\begin{array}{c} \frac{\partial }{\partial x^{+}}\nabla H\;\left(\frac{x+x^{+}}{2}\right)=\frac{\partial }{\partial x}\nabla H\;\left(\frac{x+x^{+}}{2}\right). \end{array}$$

However, invariance of *H* is lost and is violated in $O(\delta^{3})$. In fact, the expansion of the right-hand side of (55) matches the exact sampled dynamics in $O(\delta^{3})$ only.

#### 4.3.3. Higher-Order Hamiltonian Integrators

An interesting outcome of the result in Theorem 3 is the definition higher-order approximations that preserve both conservativeness and the symplectic structure. At first, a 3^*rd*^-order approximate model is defined.

**Theorem** **6.** *The discrete-time dynamics*

(56) $$\begin{array}{c} x^{+}-x=J_{3}^{\delta }\left(x\right)G\left(x,x^{+}\right)\;\mathrm{with}\;J_{3}^{\delta }\left(x\right)=\delta J_{1}+\delta^{2}J_{2}+\delta^{3}J_{3}=\delta J-\frac{\delta^{3}}{12}J{\left(H_{\mathrm{ess}}\left(x\right)J\right)}^{2} \end{array}$$

*defines a 3^rd^-order Hamiltonian integrator of the continuous-time dynamics (39) that is:*

- *exactly conservative; i.e.,*
$$\begin{array}{c} H\left(x^{+}\right)-H\left(x\right)=G{\left(x,x^{+}\right)}^{⊤}J_{3}^{\delta }\left(x\right)G\left(x,x^{+}\right)=0 \end{array}$$
*since $J_{3}^{\delta }\left(x\right)$ is skew-symmetric;*
- *symplectic in $O(\delta^{4})$; i.e.,*
$$\begin{array}{c} {\left(I+J_{3}^{\delta }G_{x}\right)}^{⊤}{\left(I-J_{3}^{\delta }G_{x^{+}}\right)}^{-⊤}J{\left(I-J_{3}^{\delta }G_{x^{+}}\right)}^{-1}\left(I+J_{3}^{\delta }G_{x}\right)=J+O\left(\delta^{4}\right). \end{array}$$
- *Moreover, the exact sampled dynamics (41) satisfy the implicit form (56) in $O(\delta^{4})$.*

**Proof.**  The result follows by comparing the expansion of the right-hand side of (56) with the exact sampled increment $F_{H}^{\delta }\left(x\right)$. Using Corollary 3, one obtains

$$\begin{array}{cc} J_{3}^{\delta }\left(x\right)G\left(x,x+F_{H}^{\delta }\left(x\right)\right) & =\delta J\nabla H\left(x\right)+\frac{\delta^{2}}{2}JH_{\mathrm{ess}}\left(x\right)J\nabla H\left(x\right)+\frac{\delta^{3}}{3!}{\left(JH_{\mathrm{ess}}\left(x\right)\right)}^{2}J\nabla H\left(x\right)\\ & +\frac{\delta^{3}}{3!}JL_{J\nabla H}H_{\mathrm{ess}}\left(x\right)J\nabla H\left(x\right)=F_{H}^{\delta }\left(x\right)+O\left(\delta^{4}\right). \end{array}$$

The dynamics are symplectic in $O(\delta^{4})$ since it matches the exact flow at the same order. □

**Remark** **20.** *We note that the dynamics are symplectic in $O(\delta^{4})$ because state matching holds in $O(\delta^{4})$. This property does not result from the structure. Substituting in (56), the discrete gradient $G(x,x^{+})$ with it midpoint approximation $\nabla H(\frac{x+x^{+}}{2})$, one obtains the dynamics*

$$\begin{array}{c} x^{+}-x=J_{3}^{\delta }\left(x\right)\nabla H\left(\frac{x+x^{+}}{2}\right) \end{array}$$

*which are not symplectic due to the x-dependency of the matrix $J_{3}^{\delta }\left(x\right)$. Conservativeness is lost also.*

The result in Theorem 6 can be generalized to higher-order approximations.

**Theorem** **7.** *The discrete-time dynamics*

(57) $$\begin{array}{c} x^{+}-x=J_{p}^{\delta }\left(x\right)G\left(x,x^{+}\right)=\sum_{i=1}^{p}\delta^{i}J_{i}\left(x\right)G\left(x,x^{+}\right) \end{array}$$

*define an approximate Hamiltonian integrator of order p of the continuous-time dynamics (39), that is,*

- *conservative in $O(\delta^{p+1})$;*
- *symplectic in $O(\delta^{p+1})$;*
- *the exact sampled dynamics (41) satisfy the implicit form (57) in $O(\delta^{p+1})$.*

We note that all the properties, conservativeness, symplectic structure and state matching, hold at the same order of approximation. However, as the provided skew-symmetry of $J_{p}^{\delta }\left(x\right)$ can be proved, then conservativeness holds globally.

In conclusion, the 3*^rd^*-order Hamiltonian integrator we define is expected to perform better than the two previously recalled approximations (conservative and implicit midpoint) since it globally ensures invariance of the Hamiltonian function and ensures state matching of the exact sampled dynamics in $O(\delta^{4})$. On the other hand, the dynamics are symplectic at the order 4 but may be deteriorated by the approximation of the discrete gradient $G(x,x^{+})$ used for computational facilities. The same difficulty affects the simulations of the conservative (54) and midpoint (55) approximations.

#### 4.3.4. The Linear Case

We then specify these approximate integrator schemes to quadratic Hamiltonian to define higher-order Hamiltonian integrators that are globally symplectic and conservative. Their explicit forms match the trajectories of the continuous-time dynamics up to increasing order in δ. Making reference to Theorem 5, and denoting by $H_{2p+1}^{\delta }\left(x\right)$ the truncation at the order 2p+1 of the sampled Hamiltonian function; i.e.,

$$\begin{array}{cc} H_{2p+1}^{\delta }\left(x\right) & =\frac{1}{2}x^{⊤}P_{2p+1}^{\delta }x=\frac{\delta }{2}P+\sum_{i=1}^{p}\frac{\delta^{2i+1}}{2}P_{2i+1}\;\mathrm{with}\;P_{1}^{\delta }=P \end{array}$$

one states the following:

**Theorem** **8.** *For p≥0, the canonical discrete-time Hamiltonian dynamics*

(58) $$\begin{array}{c} x^{+}-x=J\frac{P_{2p+1}^{\delta }}{2}\left(x+x^{+}\right) \end{array}$$

*with explicit form*

(59) $$\begin{array}{c} x^{+}={\left(I-J\frac{P_{2p+1}^{\delta }}{2}\right)}^{-1}\left(I+J\frac{P_{2p+1}^{\delta }}{2}\right)x \end{array}$$

*define a linear Hamiltonian integrator dynamic of order 2(p+1) associated with (47). It is globally conservative and symplectic. Moreover, its state trajectories match those of the continuous-time in $0(\delta^{2p+3})$.*

The result is constructive. From Corollary 4, one computes iteratively.

**Corollary** **6.** *The Hamiltonian $H_{2p+1}^{\delta }\left(x\right)=\frac{1}{2}x^{⊤}P_{2p+1}^{\delta }x$ can be iteratively computed. One gets for p≥1 with $P_{1}^{\delta }=\delta P$*

$$\begin{array}{cc} p=1: & \;\frac{1}{2}P_{3}^{\delta }\left(x\right)=\frac{1}{2}x^{⊤}\left(\delta P-\frac{\delta^{3}}{12}P{\left(JP\right)}^{2}\right)x,\\ p=2: & \;\frac{1}{2}P_{5}^{\delta }\left(x\right)=\frac{1}{2}x^{⊤}\left(\delta P-\frac{\delta^{3}}{12}P{\left(JP\right)}^{2}+\frac{\delta^{5}}{120}P{\left(JP\right)}^{4}\right)x,\\ \; & \;\vdots \\ p: & \;\frac{1}{2}P_{2p+1}^{\delta }=\frac{1}{2}P_{2p-1}^{\delta }+{\left(\frac{\delta }{2}\right)}^{2p+1}\frac{2^{2p+2}\left(2^{2p+2}-1\right)B_{2p+2}}{(2p+2)!}P{\left(JP\right)}^{2p}. \end{array}$$

From Theorem 4, one gets, equivalently to (58), the following:

**Corollary** **7.** *The canonical Hamiltonian dynamics *(58)* can be equivalently rewritten with respect to the H-function with modified interconnection matrix, so getting*

(60) $$\begin{array}{c} x^{+}-x=J_{2p+1}^{\delta }\frac{P}{2}\left(x+x^{+}\right) \end{array}$$

*with $J_{2p+1}^{\delta }=\delta J+\sum_{i=1}^{p}\delta^{2i+1}J_{2i+1}$, a skew symmetric interconnection matrix satisfying $J_{2p+1}^{\delta }P=JP_{2p+1}^{\delta }$.*

By construction, both H(x) and $H_{2p+1}^{\delta }\left(x\right)$ are constants of motions of the sampled dynamics (58) or, equivalently, (60). This is consistent with the fact that $H^{\delta }\left(x\right)\to H\left(x\right)$ as δ→0.

For completeness, the 3^*rd*^-order Hamiltonian integrator is precisely set.

**Proposition** **8.** *The discrete-time dynamics*

(61) $$\begin{array}{cc} x^{+}-x & =J_{3}^{\delta }\frac{P}{2}\left(x+x^{+}\right)=J\frac{P_{3}^{\delta }}{2}\left(x+x^{+}\right)=\left(\delta J-\frac{\delta^{3}}{12}J{\left(H_{\mathrm{ess}}\left(x\right)J\right)}^{2}\right)\frac{P}{2}\left(x+x^{+}\right) \end{array}$$

*define a 3^rd^-order Hamiltonian integrator of the continuous-time dynamics *(39)* that is globally conservative since $J_{3}^{\delta }$ is skew-symmetric and globally symplectic. The exact sampled dynamics (48) satisfies the implicit form (61) in $O(\delta^{4})$.*

**Remark** **21.** *In this linear case, the approximations defined by (54) or (55) coincide and recover the so defined first-order Hamiltonian integrator dynamics (p=0)*

$$x^{+}-x=\delta J\frac{P}{2}\left(x+x^{+}\right)\;\mathrm{or},\;\mathrm{equivalently},\;x^{+}={\left(I-\delta J\frac{P}{2}\right)}^{-1}\left(I+\delta J\frac{P}{2}\right)x.$$

In conclusion, the proposed construction yields a hierarchy of symplectic integrators of arbitrarily high order, obtained through a direct truncation of the sampled Hamiltonian, which are globally conservative, thus providing a systematic extension of the symplectic integrator or implicit midpoint schemes.

## 5. Simulated Examples

Simulations over simple examples substantially confirm the performances of the 3^*rd*^-order Hamiltonian integrator scheme proposed with respect to a first-order approximation in both linear and nonlinear contexts. Comparisons with the continuous-time dynamics are reported too. The authors have been supported by the accessible free edition of ChatGPT for writing and debugging the Python (v3.14.6) code in the reported simulations.

### 5.1. Nonlinear Case Studies

Let us first consider the two Hamiltonian functions

(62) $$\begin{array}{c} H_{P}\left(x\right)=\left(1-cosx_{1}\right)+\frac{1}{2}x_{2}^{2}\;\mathrm{and}\;H_{E}\left(x\right)=x_{1}logx_{1}-x_{1}x_{2}+\frac{1}{2}x_{2}^{2}. \end{array}$$

The first one represents the well known undamped pendulum while the second is an academic one.

According to Theorem 6, the 3^*rd*^-order Hamiltonian integrator dynamics takes the form

(63) $$\begin{array}{c} x^{+}-x=\alpha^{\delta }\left(x\right)JG\left(x,x^{+}\right) \end{array}$$

with $J_{3}^{\delta }\left(x\right)=\delta \left(I-\frac{\delta^{2}}{12}JH_{ess}\left(x\right)JH_{ess}\left(x\right)\right)J=\alpha^{\delta }\left(x\right)J$. We recall that (63) is, by construction, globally conservative and symplectic in $O(\delta^{4})$ as a consequence of state matching in $O(\delta^{4})$. Easy computations give, respectively,

(64) $$\begin{array}{cc} \nabla H_{P}\left(x\right)=\left(\begin{array}{c} sinx_{1}\\ x_{2} \end{array}\right)\; & \mathrm{and}\;\nabla H_{E}\left(x\right)=\left(\begin{array}{c} 1+logx_{1}-x_{2}\\ x_{2}-x_{1} \end{array}\right) \end{array}$$

(65) $$\begin{array}{cc} H_{P_{ess}}\left(x\right)=\left(\begin{array}{cc} cosx_{1} & 0\\ 0 & 1 \end{array}\right)\; & \mathrm{and}\;H_{E_{ess}}\left(x\right)=\left(\begin{array}{cc} \frac{1}{x_{1}} & -1\\ -1 & 1 \end{array}\right) \end{array}$$

(66) $$\begin{array}{cc} \alpha_{P}^{\delta }\left(x\right)=\delta +\frac{\delta^{3}}{12}cosx_{1}\; & \mathrm{and}\;\alpha_{E}^{\delta }\left(x\right)=\delta +\frac{\delta^{3}}{12x_{1}} \end{array}$$

so computing the discrete gradient

(67) $$\begin{array}{c} G_{P}\left(x,x^{+}\right)=\left(\begin{array}{c} \frac{cosx_{1}-cosx_{1}^{+}}{x_{1}^{+}-x_{1}}\\ \frac{x_{2}+x_{2}^{+}}{2} \end{array}\right)\;\mathrm{and}\;G_{E}\left(x,x^{+}\right)=\left(\begin{array}{c} \frac{x_{1}^{+}logx_{1}^{+}-x_{1}logx_{1}}{x_{1}^{+}-x_{1}}-\frac{x_{2}^{+}+x_{2}}{2}\\ \frac{x_{2}+x_{2}^{+}}{2}-\frac{x_{1}+x_{1}^{+}}{2} \end{array}\right). \end{array}$$

To obtain a computable explicit form, one further substitutes $G(x,x^{+})$ in (63) with its approximation

$$\begin{array}{cc} G(x,x^{+}) & =\nabla H\left(x\right)+\frac{1}{2}H_{ess}\left(x\right)\left(x^{+}-x\right)+0(|x^{+}-x{|}^{2}) \end{array}$$

so getting a computable explicit representation of (63)

(68) $$\begin{array}{cc} x^{+} & =x+\alpha^{\delta }\left(x\right){\left(I-\frac{1}{2}\alpha^{\delta }\left(x\right)JH_{ess}\left(x\right)\right)}^{-1}J\nabla H\left(x\right) \end{array}$$

that remains valid in $O(\delta^{3})$ only.

**Figure 1:**
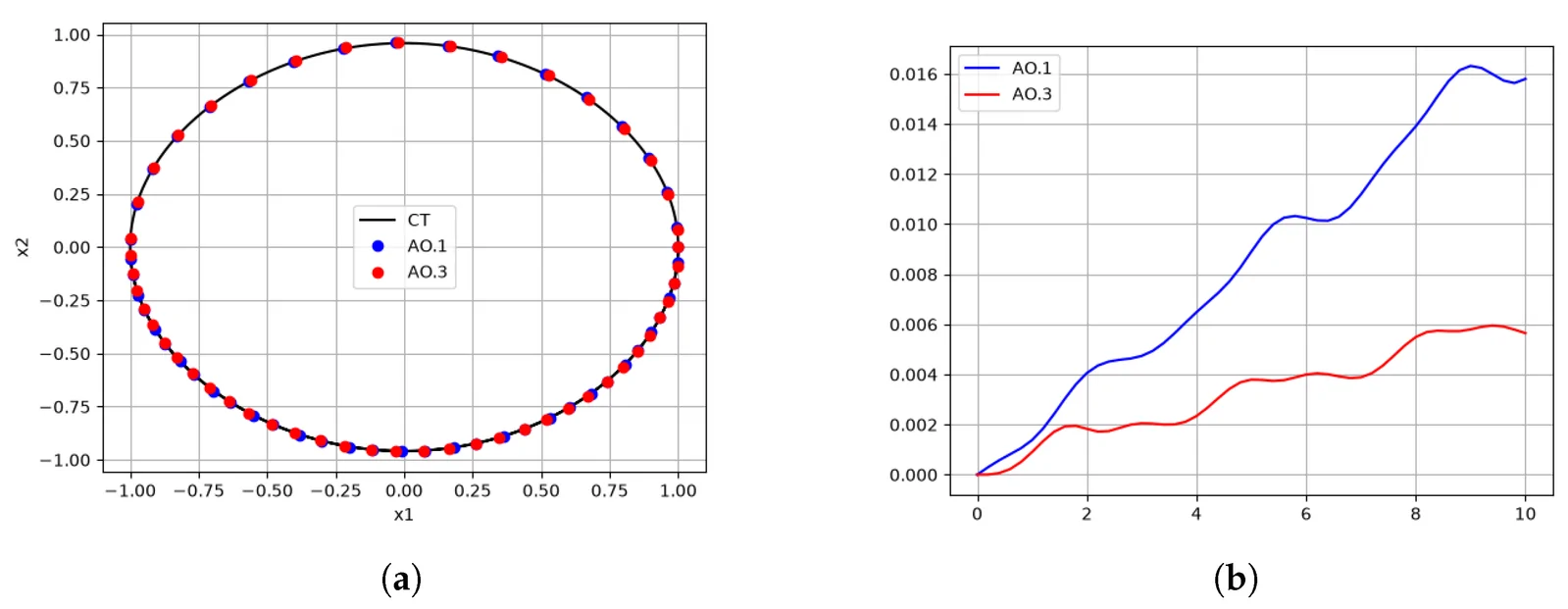
P-dynamics (δ=0.2). (**a**) State space trajectories and (**b**) state evolution errors.

**Figure 2:**
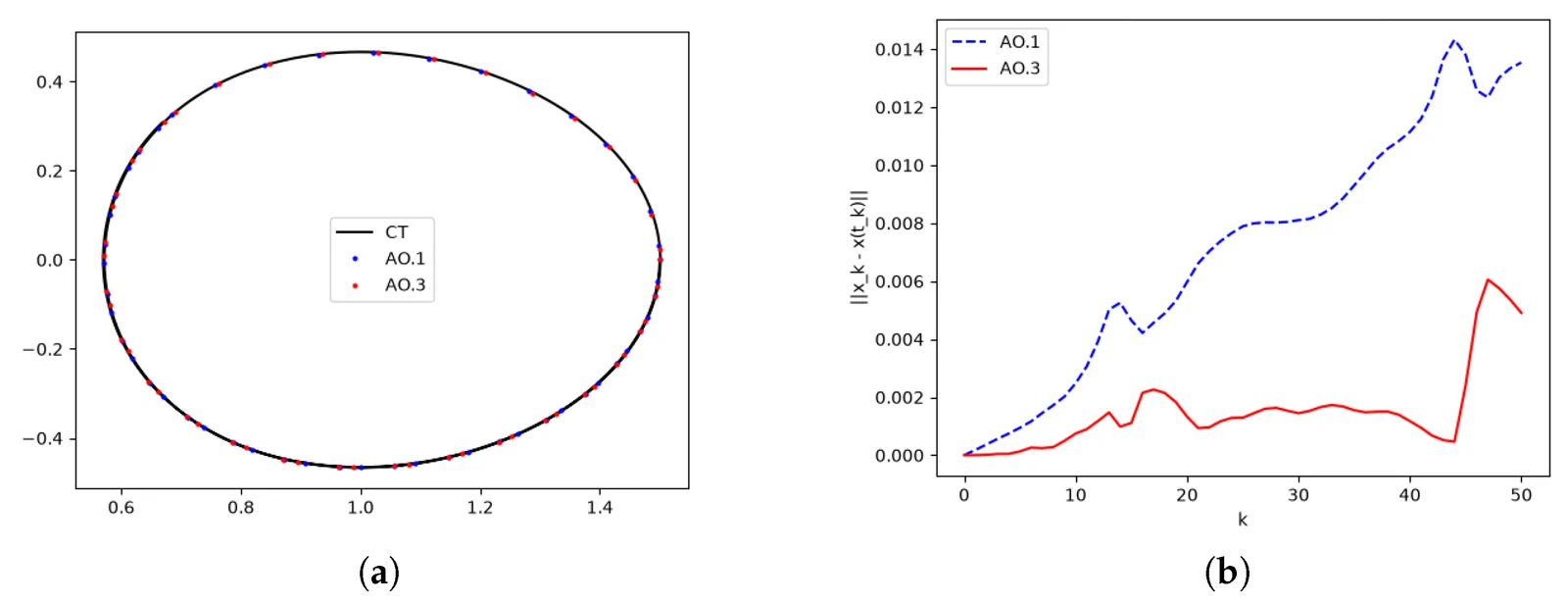
E-dynamics (δ=0.2). (**a**): State space trajectories and (**b**) state evolution errors.

**Figure 3:**
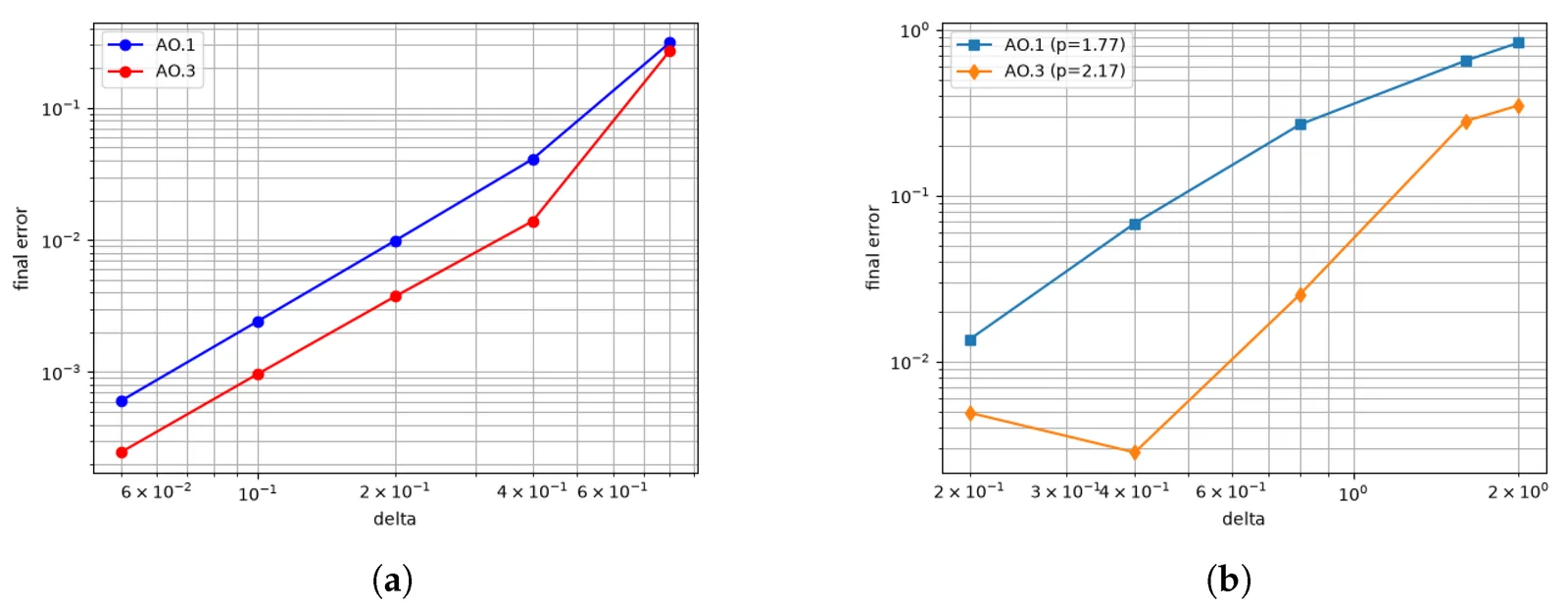
(Log–log)-convergency. (**a**): P-dynamics and (**b**): E-dynamics. For different values of δ.

Figure 1, Figure 2 and Figure 3 compare the results of the approximate explicit dynamics at the order 1 (AO.1) (when setting $\alpha^{\delta }\left(x\right)=\delta$) and at the order 3 (AO.3) for the “P-dynamics” and “E-dynamics”. The state space portrait and the errors with respect to the continuous-time evolution are in Figure 1 and Figure 2.

The reduction of the evolution errors and the numerically estimated convergence order, obtained from the (log–log) slope of the error as a function of the sampling step reported in Figure 3, confirms the performances of (AO.3).

**Figure 4:**
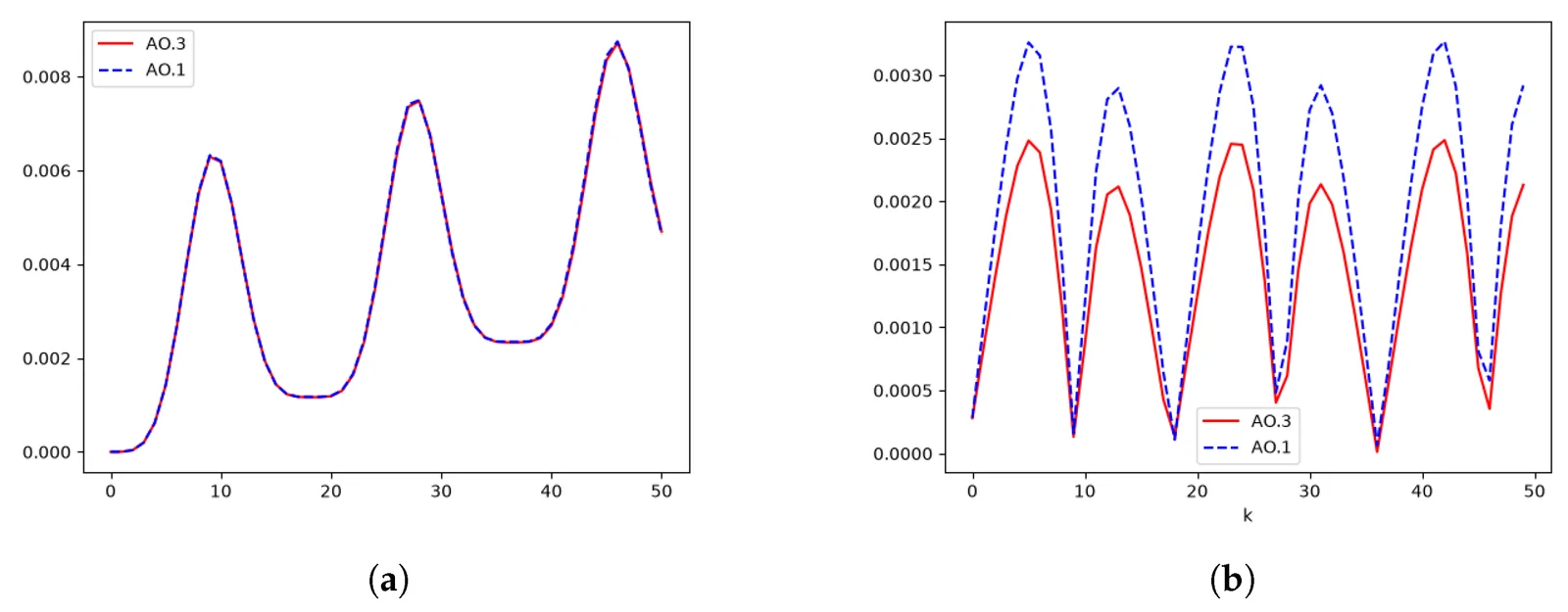
P-dynamics (δ=0.2) (**a**): Energy preservation errors and (**b**): symplectic structure errors.

**Figure 5:**
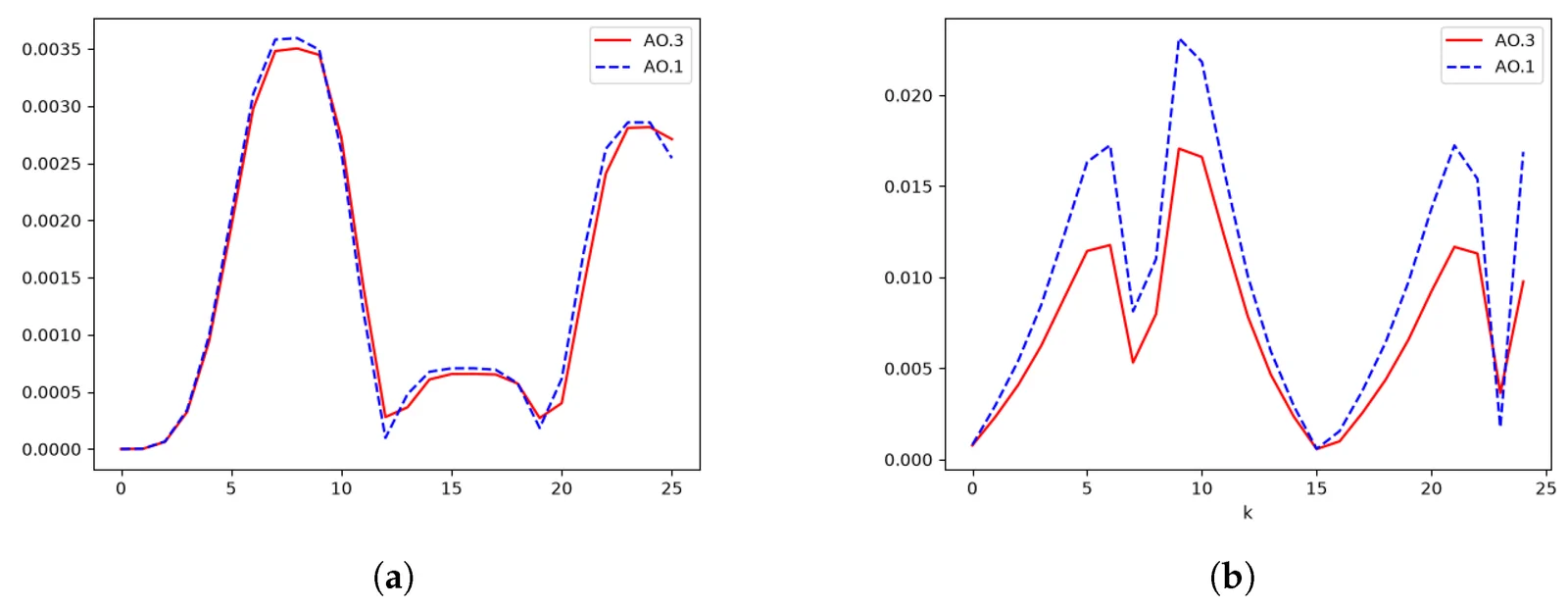
E-dynamics. (**a**): Energy preservation errors and (**b**): symplectic structure errors.

As expected, energy preservation is well satisfied by both (AO.1) and (AO.3) in Figure 4 and Figure 5a. An improvement regarding symplecticity can be observed from the curves in Figure 4 and Figure 5b that is essentially due to the reduction of the state evolution errors according to (AO.3). As a consequence, the estimated orders of approximations are as follows: for the P-dynamics 2.09 under (AO.1) and 2.41 under (AO.3) and for the E-dynamics 1.77 under (AO.1) and 2.17 under (AO.3).

### 5.2. A Linear Example

Considering now the quadratic Hamiltonian

(69) $$\begin{array}{c} H\left(x\right)=\frac{1}{2}x^{⊤}\left(\begin{array}{cc} 2 & 0.5\\ 0.5 & 1 \end{array}\right)x \end{array}$$

**Figure 6:**
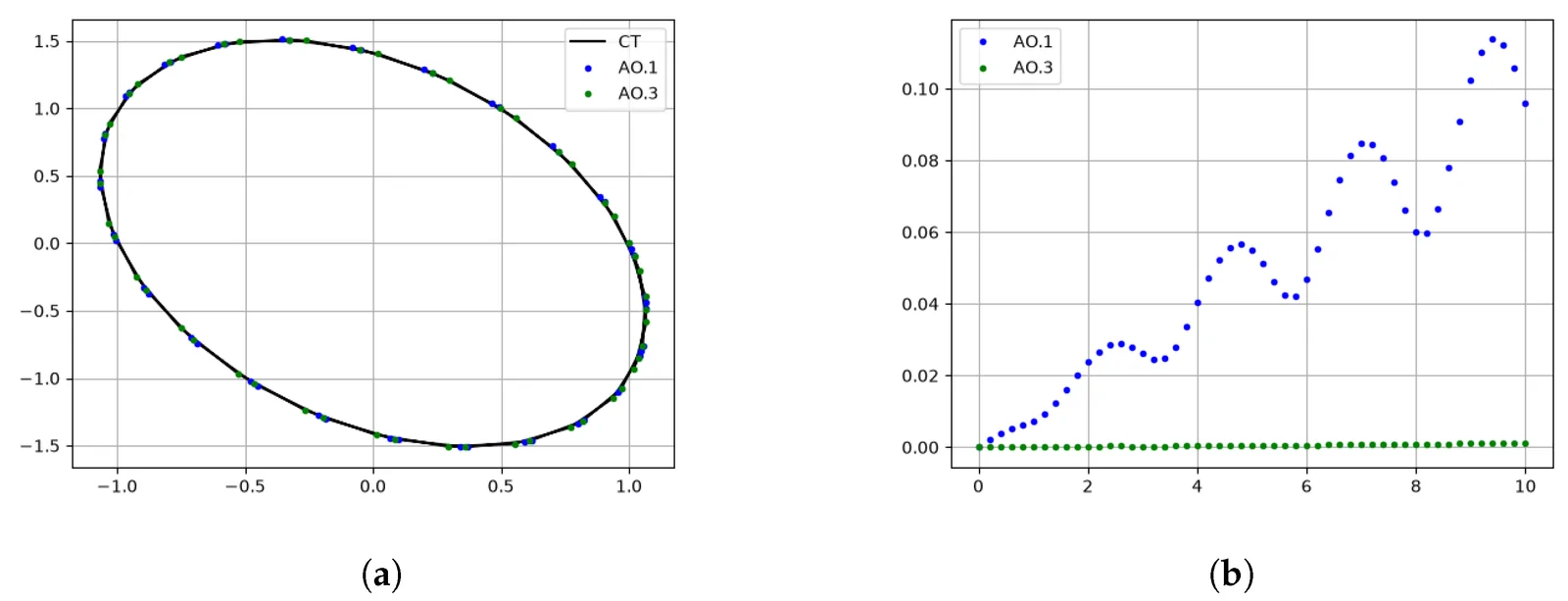
Linear AO.1 and AO.3 approximated dynamics. (**a**): State space trejectories and (**b**): state evolution errors.

Simulations are reported in Figure 6; comparing the implicit midpoint scheme (AO.1) with the third-order Hamiltonian integrator, we propose (AO.3). The results confirm that both schemes are conservative and symplectic. The estimated orders of approximation are 1.56 for (AO.1) and 2.98 for (AO.3), thus confirming the theoretical results. Simulations for Hamiltonians of orders four and six were also performed. In all cases, the estimated orders of approximation were below 2.00 for (AO.1) and close to 3.00 for (AO.3), consistent with the theoretical predictions.

## 6. Conclusions

In this paper, necessary and sufficient conditions guaranteeing a symplectic structure for a class of conservative nonlinear Hamiltonian dynamics have been established. In the linear setting, the interplay between an implicit representation described by an infinitesimally symplectic matrix and an explicit representation described by a symplectic matrix is fully clarified.

The study of Hamiltonian dynamics under sampling reveals interesting properties. Under exact sampling, the resulting dynamics does not conform to the representation proposed here, but to its generalization through a modified interconnection matrix that depends on both the state and the sampling period. Accordingly, both conservativeness and the symplectic structure are preserved. In the linear case, the exact sampled model recovers a canonical discrete-time Hamiltonian form with respect to a modified Hamiltonian function, thereby defining a sampled generalized Hamiltonian function.

Since the results are constructive and the corresponding solutions characterized through functional expansions in the sampling period, approximate representations of increasing order can be readily derived. Their properties with respect to both the conservative and symplectic properties can be explicitly characterized. A third-order symplectic and conservative Hamiltonian approximate model is constructed in both the linear and nonlinear settings. Its performances are illustrated through simulations in comparison with the standard symplectic or midpoint integrator. In the linear case, a hierarchy of approximate models that are both conservative and symplectic is proposed; each model matches the exact sampled state trajectory with an error of increasing order in the sampling period. To the best of our knowledge, similar integrator designs have not been developed in the literature. They may, therefore, open interesting perspectives in numerical analysis, provided their performances are further assessed through appropriate validation tests.

Building on our expertise and motivated by the fact that modeling is a first step towards control, a natural perspective is to extend these representations to port-controlled Hamiltonian systems, incorporating controlled dynamics and an output map. In this context, the introduced notions of generalized interconnection matrices, together with their possible extensions to generalized resistive matrices and generalized Hamiltonian functions, may provide a framework for reconsidering control by interconnection of port-Hamiltonian structures along the lines of the approaches discussed in the cited references [18,19,20,21,22,23,24,25,26]. Work is currently in progress in this direction.

## Data Availability

The original contributions presented in this study are included in the article. Further inquiries can be directed to the corresponding author.

## Appendix A

**Lemma** **A1.**

- *Any infinitesimally symplectic matrix $L\in R^{2n}\times R^{2n}$ can be written as*
  (A1)
  $$\begin{array}{c} L=JS \end{array}$$
  *for a suitably defined symmetric matrix S.*
- *The matrix*
  (A2)
  $$\begin{array}{c} M={\left(I-JA\right)}^{-1}\left(I+JB\right) \end{array}$$
  *with A and B symmetric without eigenvalues in *1*, is symplectic if and only if*
  AJA=BJB.

**Proof.** 

- Note first that any L=JS, with *S* symmetric, satisfies (A1); i.e., $JL+L^{⊤}J=0$. On the other hand, $JL+L^{⊤}J=0$ implies symmetry of −JL as $L^{⊤}J=-JL$, from which *L* can be rewritten as L=J(−JL)=JS with symmetric S=−JL.
- From the condition of symplecticity
  $${\left(I+JB\right)}^{⊤}{\left(I-JA\right)}^{-⊤}J{\left(I-JA\right)}^{-1}\left(I+JB\right)=J$$
  on the domains of invertibility of the matrices involved, the previous equality rewrites
  $${\left(I-JA\right)}^{-⊤}J{\left(I-JA\right)}^{-1}={\left(I+JB\right)}^{-⊤}J{\left(I+JB\right)}^{-1}$$
  that is also equivalent to
  $$\left(I-JA\right)J{\left(I-JA\right)}^{⊤}=\left(I+JB\right)J{\left(I+JB\right)}^{⊤}.$$
  Note that with *A* and *B* symmetric, one has
  $${\left(I-JA\right)}^{⊤}=I+AJ\;and\;{\left(I+JB\right)}^{⊤}=I-BJ$$
  so that the condition of symplecticity rewrites
  J+JAJ−JAJ−JAJAJ=J−JBJ+JBJ−JBJBJ
  that is equivalent to
  AJA=BJB.  □
